# Evolution and functional role prediction of the CYP6DE and CYP6DJ subfamilies in *Dendroctonus* (Curculionidae: Scolytinae) bark beetles

**DOI:** 10.3389/fmolb.2023.1274838

**Published:** 2023-10-09

**Authors:** J. Manuel Quijano-Barraza, Gerardo Zúñiga, Claudia Cano-Ramírez, María Fernanda López, Gema L. Ramírez-Salinas, Moises Becerril

**Affiliations:** ^1^ Laboratorio de Variación Biológica y Evolución, Departamento de Zoología, Escuela Nacional de Ciencias Biológicas, Instituto Politécnico Nacional, Prolongación de Carpio y Plan de Ayala s/n, Mexico City, Mexico; ^2^ Laboratorio de Modelado Molecular y Diseño de Fármacos, Departamento de Bioquímica, Escuela Superior de Medicina, Instituto Politécnico Nacional, Mexico City, Mexico

**Keywords:** cytochrome P450, detoxification, *dendroctonus*, functional divergence, protein-ligand docking, birth-death model

## Abstract

*Dendroctonus*-bark beetles are natural components and key ecological agents of coniferous forests. They spend most of their lives under the bark, where they are exposed to highly toxic terpenes present in the oleoresin. Cytochrome P450 (CYP) is a multigene family involved in the detoxification of these compounds. It has been demonstrated that CYP6DE and CYP6DJ subfamilies hydroxylate monoterpenes, whose derivatives can act as pheromone synergist compounds or be pheromones themselves in these insects. Given the diversity and functional role of CYPs, we investigated whether these cytochromes have retained their function throughout the evolution of these insects. To test this hypothesis, we performed a Bayesian phylogenetic analysis to determine phylogenetic subgroups of cytochromes in these subfamilies. Subgroups were mapped and reconciled with the *Dendroctonus* phylogeny. Molecular docking analyses were performed with the cytochromes of each subgroup and enantiomers of *α*-pinene and *β*-pinene, (+)-3-carene, *β*-myrcene and *R*-(+)-limonene. In addition, functional divergence analysis was performed to identify critical amino acid sites that influence changes in catalytic site conformation and/or protein folding. Three and two phylogenetic subgroups were recovered for the CYP6DE and CYP6DJ subfamilies, respectively. Mapping and reconciliation analysis showed different gain and loss patterns for cytochromes of each subgroup. Functional predictions indicated that the cytochromes analyzed are able to hydroxylate all monoterpenes; however, they showed preferential affinities to different monoterpenes. Functional divergence analyses indicated that the CYP6DE subfamily has experimented type I and II divergence, whereas the CYP6DJ subfamily has evolved under strong functional constraints. Results suggest cytochromes of the CYP6DE subfamily evolve to reinforce their detoxifying capacity hydroxylating mainly *α*- and *β*-pinene to (+) and (−)-*trans*-verbenol, being the negative enantiomer used as a pheromone by several *Dendroctonus* species; whereas cytochromes of the CYP6DJ subfamily appear to retain their original function related to the detoxification of these compounds.

## 1 Introduction

*Dendroctonus* bark beetles (Curculionidae: Scolytinae) are natural components and key ecological agents of conifer forests in North and Central America, and Eurasia, as they participate in essential ecological processes, such as nutrient cycling, forest succession, and watershed regulation by the removal of old, damaged, diseased, or weakened trees (Raffa et al., 2015). Yet, they are also considered disturbance agents because some species produce extensive outbreaks which affect forest structure and landscape, biodiversity, recreation sites, and property values (Grégoire et al., 2015).

The life cycle of these bark beetles begins when the females detect volatile terpenes released by the trees, which serve as primary attractants (kairomones) to select susceptible trees. Once in the tree, females bore into the phloem, release pheromones and compounds derived from terpene metabolism to attract males. Thereafter, both sexes copulate and excavate galleries along which the females oviposit. For a successful colonization, kairomones and pheromones integrate specific cues that facilitate mass attacks on trees. The larvae feed on phloem and develop as they construct galleries that end in pupal chambers, from which brood adults emerge. During the colonization, they must overcome the defensive mechanisms of host trees, especially the chemical constitutive and induced defenses integrated by monoterpenes (10 carbon atoms), non-volatile diterpenes (20 carbon atoms), sesquiterpenes (15 carbon atoms), and phenolic compounds present in the resin (Krokene, 2015).

These terpenes are toxic to bark beetles and their symbionts, which can inflict severe damage to membranous cellular structures and even result in death (López et al., 2011; Chiu et al., 2017). The successful colonization of trees by these beetles, partly depends on the evolution of enzymatic complexes, such as glutathione-S-transferases, carboxylesterases, and cytochromes P450, which metabolize these compounds (Li et al., 2007; Blomquist et al., 2021; Dai et al., 2021; Torres-Banda et al., 2022). Cytochromes P450 (CYPs) catalyze reactions of oxidation, epoxidation, dehydrogenation, hydrolysis, and reduction. The CYPs evolution in herbivorous insects is characterized by their catalytic versatility and substrate specificity, which is associated with the diversity of phytochemicals present in host plants (Schuler and Berenbaum, 2013; Sezutsu et al., 2013; Rane et al., 2019; Lu et al., 2021).

Bark beetle genomes and transcriptomes have shown a wide diversity of cytochromes P450 (Keeling, 2016; Powell et al., 2021; Liu et al., 2022), which present significant transcriptional activity after insects are fed or stimulated with terpenoids compounds (Torres-Banda et al., 2022). Differential and heterologous expression studies have demonstrated the induction and participation of specific CYP in the transformation of compounds, such as fatty acid and cuticular hydrocarbons (Ginzel et al., 2021; Nauen et al., 2021), and bicyclic monoterpenes (e.g., *α*-pinene, *β*-pinene, (+)-3-carene, *β*-myrcene, and *R*-(+)-limonene) of host trees (Blomquist et al., 2021; Torres-Banda et al., 2022). Hydroxylated enantiomers derived from these reactions can act as pheromones in some species of the genera *Dendroctonus* and *Ips*. For example, cytochromes CYP9T2 and CYP9T3 from *Ips pini* and *I. confusus* hydroxylate myrcene (Sandstrom et al., 2006) to (*R*)-(−)-ipsdienol, the main pheromone in *Ips* spp (Song et al., 2013; Blomquist et al., 2021). The CYP6DE1 of *Dendroctonus ponderosae* converts terpenes (+)- and (−)-*α*-pinene, (+)- and (−)-*β*-pinene, and (+)-3-carene into different hydroxylated enantiomers, of which only (−)-*trans*-verbenol, derived from (−)-*α*-pinene, is a pheromone in this species (Chiu et al., 2019a; Chiu et al., 2019b).

Given the diversity and functional role of CYPs in bark beetles, we hypothesized that cytochromes of CYP6DE and CYP6DJ subfamilies are involved in monoterpenes hydroxylation retain their ability to transform these compounds, regardless of whether they are involved in another function or exist other CYPs with co-responsibilities of participating in terpenes detoxification. Experimental evidence supports the participation of both subfamilies in the detoxification process and pheromone biosynthesis in *Dendroctonus*-bark beetles (Cano-Ramírez et al., 2013; López et al., 2013; Obregón-Molina et al., 2015; Nadeau et al., 2017; Chiu et al., 2019a; Chiu et al., 2019b; Sarabia et al., 2019; Liu et al., 2022; Liu and Chen, 2022; Torres-Banda et al., 2022). Thereby, to test this hypothesis, and using available genomic and transcriptomic resources of these bark beetles, we performed a molecular docking analysis with cytochromes of CYP6DE and CYP6DJ subfamilies and some monoterpenes present in host trees: (+)- and (−)-*α*-pinene, (+)- and (−)-*β*-pinene, (+)-3-carene, *β*-myrcene and *R*-(+)-limonene. In addition, based on the molecular interaction of these terpenes and CYPs, we predicted the conformational affinity between them. Lastly, we performed phylogenetic reconstruction, species-gene reconciliation, and functional divergence analyses to gain insight about the evolution and changes in the functional constraints of these subfamilies based on available data from *Dendroctonus* bark beetles.

## 2 Materials and methods

### 2.1 Protein sequence retrieval and *in silico* analysis

Full-length Cytochrome P450 sequences from the CYP6 family of the species *D. valens*, *D. rhizophagus*, *D. armandi*, and *D. ponderosae* used in this study were downloaded from the NCBI GenBank (https://www.ncbi.nlm.nih.gov/, RRID:SRC_006472). Full-length Cytochrome P450 sequences from the CYP6 family of *D. mexicanus*, *D. frontalis* and *D. adjunctus* were retrieved by an exhaustive search against the transcriptome assemblies of these species, which was carried out by the tBLASTn and tBLASTx with E-value cutoff ≤10^−5^ using the orthologs full-length sequences of CYP6DE and CYP6DJ family of species from insects mentioned above. Putative CYPs proteins were manually annotated to confirm their identity following a two-step strategy: 1) putative CYP transcripts were submitted to a BLASTp analysis against the NCBI database to determine the reference sequence with the highest identity percentage; 2) The transcripts and their corresponding reference sequence were aligned in Clustal X v.2.0 (Larkin et al., 2007, RRID:SCR_017055) to compare length, open reading frame (ORF) and untranslated regions (UTR). Redundant and chimera sequences were discarded (Supplementary Table S1).

The molecular mass (Da) and isoelectric point (pI) of the cytochromes from CYP6DE and CYP6DJ were determined with ProtParam (Gasteiger et al., 2005, RRID:SCR_018087), and their predicted subcellular localization was inferred with TargetP v.2.0 (Almagro-Armenteros et al., 2019a, RRID:SCR_019022), and DeepLoc v.2.0 (Thumuluri et al., 2022). The signal peptide region was predicted in the SignalP v.5.0 platform (Almagro-Armenteros et al., 2019b, RRID:SCR_015644).

### 2.2 Secondary structure prediction

Secondary structure elements and substrate recognition sites (SRS) of the cytochromes CYP6DE and CYP6DJ subfamilies were determined based on the cytochromes crystal structure CYP3A5 (PDB ID: 7sv2) and CYP3A4 (PDB ID: 2v0m) from *Homo sapiens*, respectively. Crystal sequences were downloaded from the RCSB-PDB (https://www.rcsb.org/, RRID:SCR_012820). The overlapping between CYPs analyzed and crystal structures had ∼30% of identity and a coverage ∼95% in all comparisons. Prediction of secondary structure elements such as *a*-helix and *ß*-sheet, and sequences alignment were performed in the ESPript v.3.0 platform (Robert and Gouet, 2014). Based on this alignment, we identify substrate recognition sites (SRS) and conserved CYP motifs (PERF, K-helix and heme-binding site) in isoforms of CYP6DE and CYP6DJ subfamilies. CYP motifs were manually located based on available information from the CYP6DJ2 orthologous (Cano-Ramírez et al., 2013; López et al., 2013) and CYP2 cytochromes (Gotoh, 1992). The domains of CYP6DE and CYP6DJ subfamilies were identified using InterPro (https://www.ebi.ac.uk/interpro/, RRID:SCR_006695).

### 2.3 Phylogenetic analysis

Amino acid sequences of CYP6DE and CYP6DJ subfamilies, along with those of CYP6, CYP9 and CYP345 families from nine species belonging to curculionids, cerambycids, tenebrionids, and chrysomelids, were aligned in Clustal X v.2.0 using default parameters for the gap opening and extension (Supplementary Table S1). Bayesian inference (BI) was performed in BEAST2 v.2.5 (Bouckaert et al., 2019, RRID:SCR_017307) using as priors the option estimated parameters, amino acid substitution model BLOSUM62 and a birth-death model. Three Markov chains independent were run for 10,000,000 million generations, sampling every 10,000. Tracer v.1.7.2 (Rambaut et al., 2018, RRID:SCR_019121) was used to check for trace convergence and values of effective sample size. After discarding the first 10% of sampled trees as burn-in using LogCombiner (Bouckaert et al., 2019), we used TreeAnnotator (Bouckaert et al., 2019) to summarize the trees distribution and values of Bayesian posterior probability in a Maximum clade credibility tree. The cytochromes of CYP6, CYP9, and CYP345E families from coleopterans species mentioned above were used as outgroups. Phylogenetic groups obtained from this analysis were identified with a letter. The name assigned to the P450 cytochromes and their variants by the P450 Nomenclature Committee (Nelson et al., 1996) was maintained. For cytochromes not yet named by this Committee, but grouped in one of the phylogenetic groups, their name was established assuming that they belonged to these phylogenetic groups; the variant number was arbitrary (Supplementary Table S1).

### 2.4 Phylogenetic instability

A phylogenetic instability analysis was performed in MiPhy v1.1.2 (Curran et al., 2018) to infer the evolutionary history of cytochromes from the CYP6DE and CYP6DJ subfamilies of the genus *Dendroctonus*. The CYP6DE and CYP6DJ phylogenies were inferred with BEAST2 v.2.5 as was described in Section 2.3. The *Dendroctonus* phylogeny was inferred by Maximum Likelihood using the cytochrome oxidase subunit-I (COI) sequences reported in Víctor and Zúñiga. (2016) (Supplementary Table S1). The analysis was performed with PhyML v.3.0 (Guindon et al., 2010, RRID:SCR_014629) and the Smart Model Selection software (SMS) (Lefort et al., 2017) in the ATGC Montpellier Bioinformatics platform (http://www.atgc-montpellier.fr/, RRID:SCR_002917). The best nucleotide evolution model was GTR + G, gamma = 0.198 according to the Akaike information criterion (-lnL = 3531.04832, AIC = 7074,55080). The reliability of the tree was evaluated with a bootstrap after 1000 permutations.

### 2.5 Molecular docking analysis

Three-dimensional models of CYP6DE and CYP6DJ cytochrome sequences were generated by homology in the SWISS-MODEL platform (https://swissmodel.expasy.org/, RRID:SCR_018123). The crystalized structure of the CYP3A subfamily from *Homo sapiens* was used as template. The models were selected and validated with Ramachandran Plot in SWISS-MODEL and ERRAT in the Structural Analysis and Verification Server (SAVES) (Colovos and Yeates, 1993, RRID:SCR_018219) (Supplementary Table S2). Pairwise topological similarities and differences among models of cytochromes of CYP6DE and CYP6DJ subfamilies and the crystallized structures of CYP3A4 and CYP3A5 proteins were evaluated across a pairwise TM-score (https://www.Zhanggroup.org/TM score/, RRID:SCR_024390). The CYP isoforms with the highest and lowest TM-score of cytochromes CYP6DE and CYP6DJ subfamilies were overlapped with the CYP3A4 and CYP3A5 crystallized proteins, respectively in PyMOL v.2.5 (Lilkova et al., 2015, RRID:SCR_000305).

Molecular docking analyses were used to evaluate most stable binding interaction between the three-dimensional models of each receptor (Supplementary Table S2) and the ligands (+)- and (−)-*α*-pinene, (+)- and (−)-*β*-pinene, (+)-3-carene, *R*-(+)-limonene, and *β*-myrcene. Ligands were obtained from PubChem (https://pubchem.ncbi.nlm.nih.gov/, RRID:SCR_004284). Receptors and ligands were optimized using the UCSF Chimera software v.1.16 (Pettersen et al., 2004, RRID:SCR_004097). The parameters included in these analyses were: 100 generations using the Lamarckian genetic algorithm (LGA), population size of 100, maximum number of evaluations of 10,000,000, maximum generations number of 27,000, gene mutation rate of 0.2, and crossover rate of 0.8 in Autodock Tools in MGL Tools v.1.5.6 Suite (Sanner, 1999). Blind dockings on each of the cytochromes and ligands were performed in Autodock v.4.2 (Morris et al., 2009, RRID:SCR_012746). The selection of conformations was performed based on the following criteria: the frequency of the receptor-ligand complex, the binding energy, and the presence of the catalytic site in the interaction. The distance between the Fe ion of the heme group and the oxygen-containing carbons of the monoterpenes was measured and displayed in 3D using PyMOL v.2.5 (Lilkova et al., 2015, RRID:SCR_000305).

A terpene was considered as a substrate of the cytochromes in molecular interaction analyses if two criteria were achieved, namely: 1) if there was interaction between the terpene and the catalytic site (heme group) and 2) if the distance between the Fe ion and the carbon atom (Fe-C distance) was <6 Å (Prasad et al., 2007). The distance was measured towards the carbon capable of being oxygenated in each monoterpene, resulting in a well-known product. For (+)- and (−)-*α*-pinene were the carbon 4 (C_4_) and the carbon methyl group (C_met_), whose hydrolysis give origin to verbenol and myrtenol, respectively; for (+)- and (−)-*β*-pinene the C_2_ and C_met_, that produce a *β*-pinene intermediary epoxide and myrtenol, respectively (Ishida, 2005); for limonene the C_4_ and C_7,_ that generate isopiperitenol and carveol, respectively; and for *β*-myrcene the C_4,_ whose hydroxylation produces ipsdienol (Sandstrom et al., 2006). The hydroxylation products of (+)-3-carene are unknown in insects, but in mammals occurs at C_10_ (Ishida, 2005); thereby the distance in this study was measured between the closest carbon to Fe ion and C_10_.

### 2.6 Functional divergence analysis

To test functional divergence after gene duplication events in isoforms of CYP6DE and CYP6DJ subfamilies, we performed type I and type II analyses based on the maximum likelihood method developed by Gu. (1999); Gu. (2003) in DIVERGE v.3.0 (Gu et al., 2013). This method estimates significant change in the evolution rate after the emergence of two paralogous sequences. Type-I analysis represents amino acids that are highly conserved in one duplicate sequences cluster, which could be highly variable in other clusters whose amino acids sites might have experienced shifted in their functional constraints. Type-II analysis evaluate evolutionary changes in the duplicated genes when the amino acid sites are under similar functional constraints in pairwise clusters, but the selected amino acid properties are different between them. Divergence coefficients (θ_I_, θ_II_) significantly greater than 0, indicate the occurrence of functional divergence.

To calculate the θ_I_ and θ_II_ divergence coefficients of phylogenetic subgroups from CYP6DE and CYP6DJ subfamilies, we analyzed the phylogenetic trees obtained from each subfamily in section 2.3. To define residues as divergence-related sites, we calculated the posterior probability value (Qk) considering a cutoff >0.7 for the type-I and type-II analyses (Knudsen et al., 2003; Gu et al., 2013).

## 3 Results

### 3.1 Analysis of full-length cytochrome CYP6DE and CYP6DJ

Amino acid sequences of cytochromes from CYP6DE subfamily varied from 475 to 508 residues, the predicted molecular mass from 54 to 58 kDa, and the isoelectric point from 8.26 to 9.26. Respect to cytochromes from CYP6DJ subfamily, these varied from 502 to 507 residues, their predicted molecular mass from 57 to 58 kDa, and the isoelectric point from 8.7 to 9.26. The predicted sub-cellular location of all analyzed cytochromes of both subfamilies showed a microsomal signal peptide of approximately 20 hydrophobic residues that are likely membrane anchors in the endoplasmic reticulum (Supplementary Table S3).

### 3.2 Secondary structure of the cytochromes CYP6DE and CYP6DJ

The secondary structure of the isoforms of CYP6DE and CYP6DJ subfamilies of *Dendroctonus* spp. were integrated by 17 *a*-helices and nine *ß*-folded (Supplementary Figures S1, S2). The characteristic motifs for CYP6DE and CYP6DJ were, respectively: PERF motif (PXRX) (positions 395-398 aa and 389-392 aa), K-helix (positions 341-344 aa and 340-343 aa), and heme-binding site (FXXGXRXCXG) (positions 413-422 aa in both subfamilies). In addition, six substrate recognition sites (SRSs) for both subfamilies were found, respectively: SRS1 (positions 81-103 aa and 80-102 aa), SRS2 (positions 183-192 aa and 182-191 aa), SRS3 (positions 217–225 aa and 216-223 aa), SRS4 (positions 273-291aa and 272-290 aa), SRS5 (positions 346-356 aa and 345-355 aa), and SRS6 (positions 454-461 aa in both subfamilies) (Supplementary Figures S1, S2). Further, the domains IPR001128-cytochrome P450, IPR002401-cytochrome P450 E-class group I, IPR036396-cytochrome P450 superfamily, and IPR017072-cytochrome P450 conserved site, were identified.

### 3.3 Phylogenetic analysis of cytochromes from CYP6 family

**FIGURE 1:**
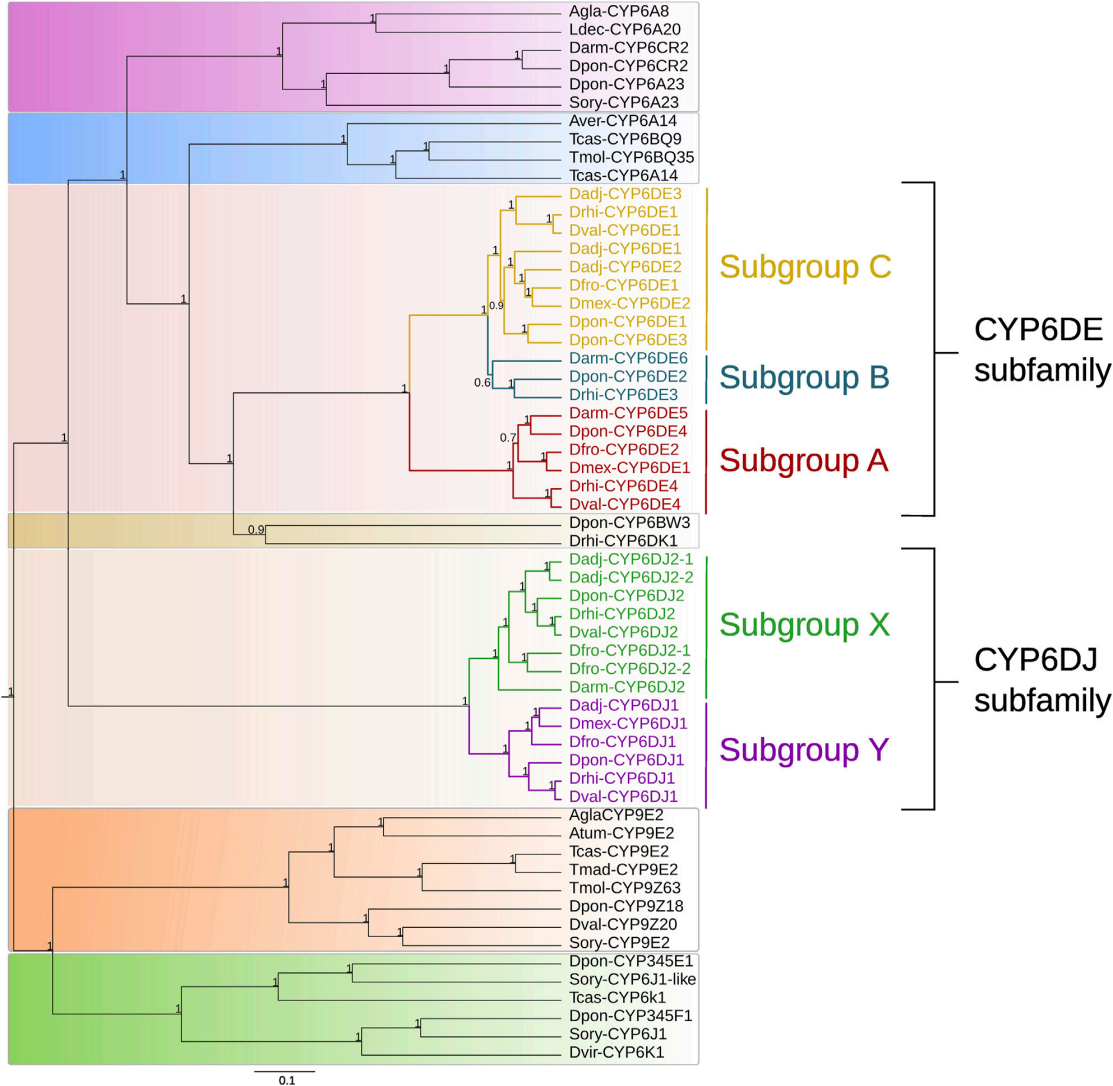
Phylogeny obtained of some CYP6 family proteins based on a Bayesian inference analysis. The subgroups from the CYP6DE subfamily are indicated as “A”, “B”, “C”, and the subgroups belonging to the CYP6DJ subfamily are marked as “X” and “Y”. Priors used were BLOSUM62 as the best amino acid substitution model and a birth-death model. The numbers indicate Bayesian posterior probabilities.

The BI analysis from sequences of different subfamilies showed the formation of seven well defined groups (Figure 1). The first integrated by CYP6A and CYP6CR subfamilies sequences from *Anoplophora glabripennis*, *Leptinotarsa decemlineata*, *Sitophilus oryzae*, *D. armandi*, and *D. ponderosae*; the second by CYP6A and CYP6BQ subfamilies sequences from *Asbolus verrucosus, Tribolium castaneum* and *Tenebrio molitor*; the third only by CYP6DE subfamily sequences from *Dendroctonus* species; the fourth by cytochromes CYP6BW3 and CYP6DK1 from *D. ponderosae* and *D. rhizophagus*; the fifth by CYP6DJ subfamily sequences exclusively from *Dendroctonus* species; the sixth by CYP9E2 isoform, and CYP9Z subfamily sequences from *A. glabripennis*, *Aethina tumida*, *T. castaneum*, *T. madens*, *T. molitor, D. ponderosae*, *D. valens,* and *S. oryzae*. Lastly, the seventh group was integrated by CYP345E, CYP6J1 and CYP6K1 isoforms from *D. ponderosae*, *S. oryzae*, *T. castaneum* and *Diabrotica virgifera* (Figure 1).

In particular, the CYP6DE subfamily from *Dendroctonus* species was integrated in three subgroups (hereafter referred to as A, B, C) (Figure 1). Subgroup A consisted of isoforms identified as Dmex-CYP6DE1, Dfron-CYP6DE2, Dpon/Drhi/Dval-CYP6DE4, and Darm-CYP6DE5; subgroup B by isoforms designated as Dpon-CYP6DE2, Drhi-CYP6DE3, and Darm-CYP6DE6, and subgroup C by isoforms labeled as Dadj/Dfron/Dpon/Drhi/Dval-CYP6DE1, Dadj/Dmex-CYP6DE2, and Dadj/Dpon-CYP6DE3. Likewise, within the fifth group integrated by sequences from the CYP6DJ subfamily from these bark beetles, two subgroups were evident (hereafter referred to as X, Y). Subgroup X was integrated by CYP6DJ2 isoforms identified as Darm/Dfron/Dpon/Drhi/Dval-CYP6DJ2, Dadj/Dfron-CYP6DE1 and 2; and subgroup Y by isoforms label as Dadj/Dfro/Dmex/Dpon/Drhi/Dval/-CYP6DJ1 (Figure 1).

**FIGURE 2:**
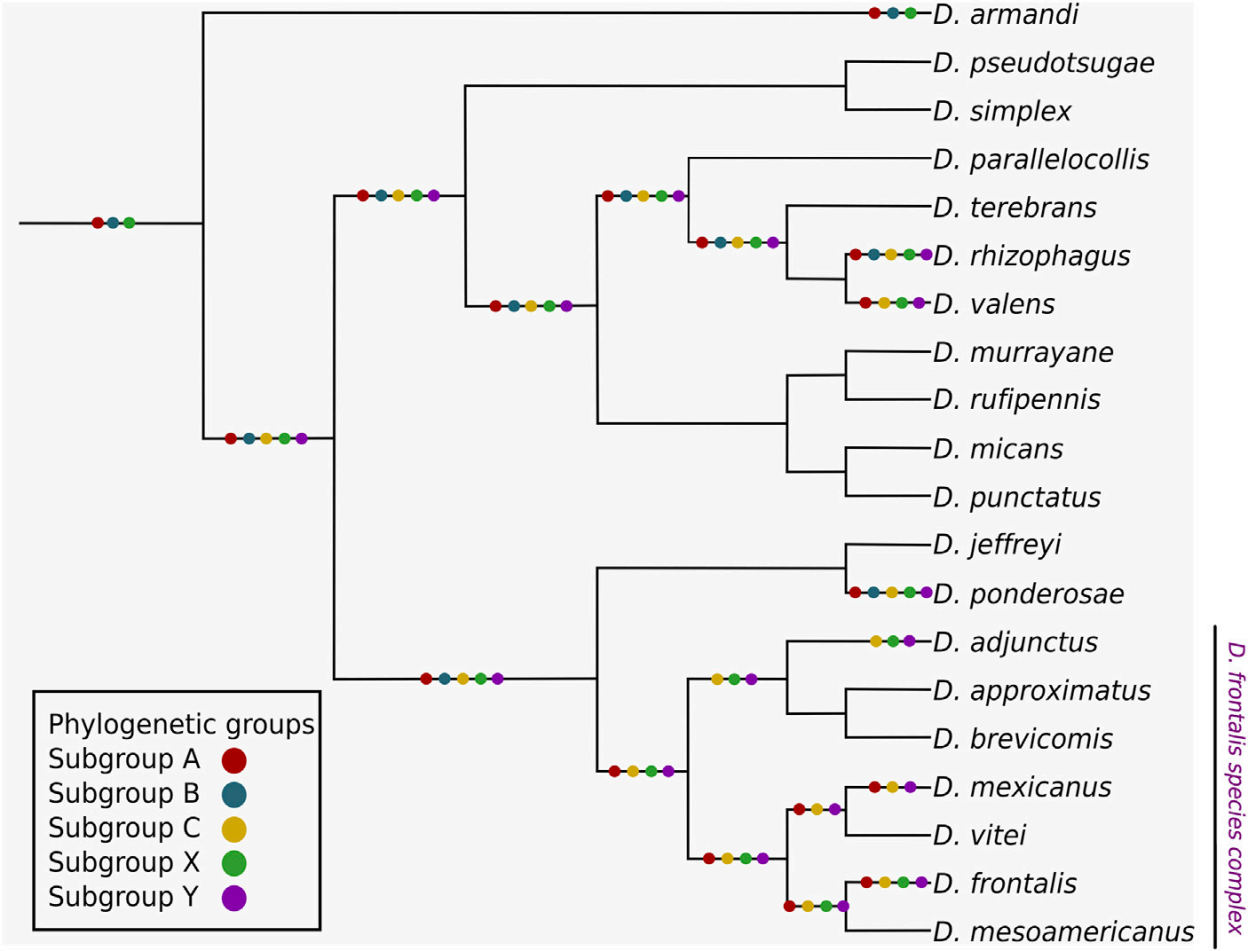
Mapping of the CYP6DE and CYP6DJ phylogenetic subgroups over a schematic representation of the *Dendroctonus* genus, the phylogenetic sub-groups are indicated with colored dots over both the terminals and common ancestor branches when at least one isoform from the corresponding subgroup is present.

The mapping of subgroups from CYP6DE and CYP6DJ subfamilies in the *Dendroctonus* phylogeny showed that subgroups A, B and X were “founding” or “ancestral”, because of their presence in *D. armandi*, the species that diverged first in this bark beetle group (Figure 2). In addition, it was also evident that subgroups C and Y originated in *Dendroctonus* species after the divergence of *D. armandi*. These subgroups were retained in most of the *Dendroctonus* species analyzed, except subgroup B that was lost in *D. valens* and in the most recent common ancestor (MRCA) from *D. frontalis* complex species. The mapping of subgroups A, C, X, and Y within this complex showed a different evolutionary history, because these were retained in *D. frontalis*, subgroup A was lost in *D. adjunctus*, and subgroup X was missing in *D. mexicanus* (Figure 2).

### 3.4 Phylogenetic stability analysis

**FIGURE 3:**
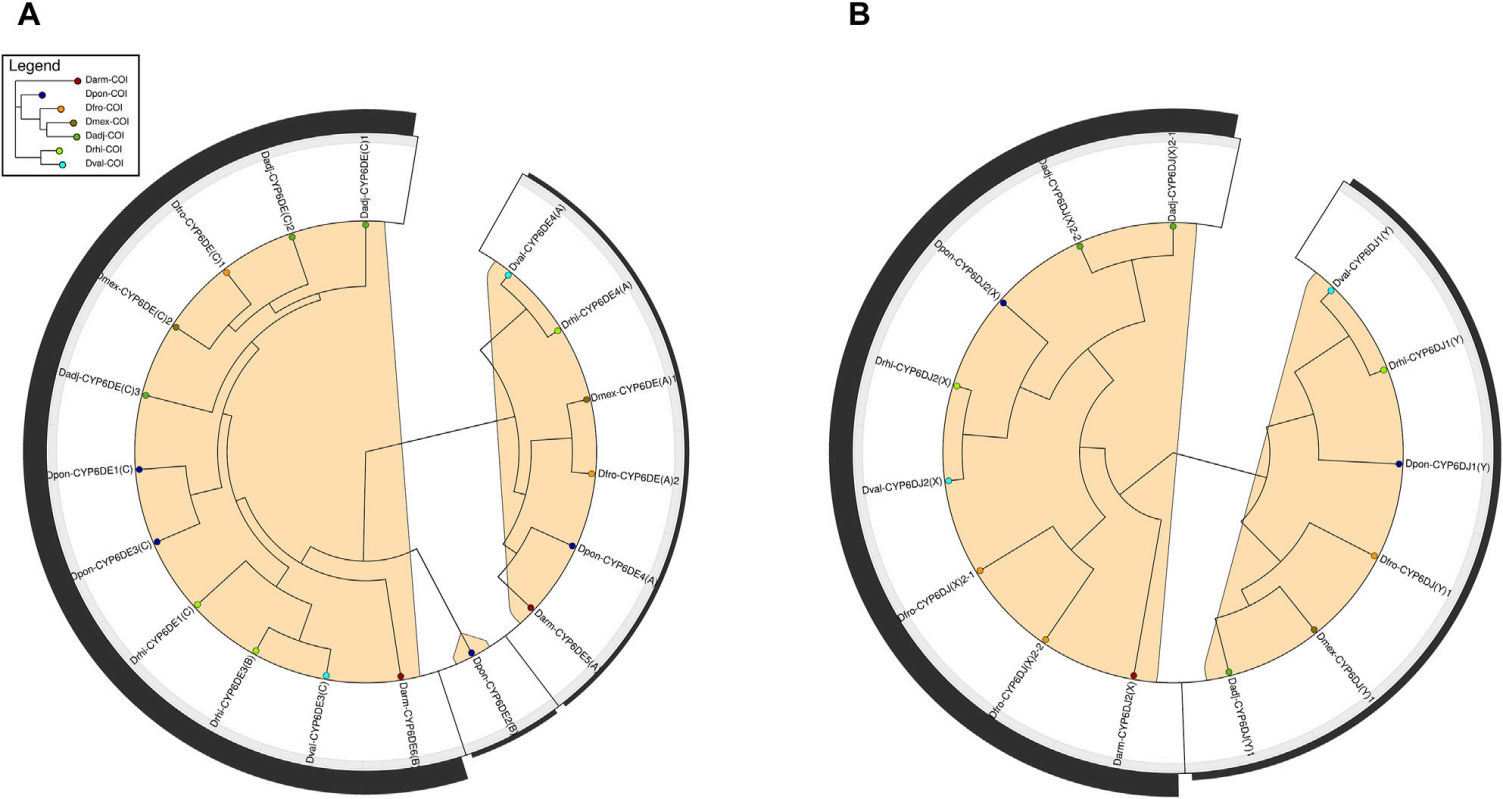
Phylogenetic reconciliation analysis of *Dendroctonus*-species *versus* CYP6DE and CYP6DJ subfamilies isoforms. **(A)**. Minimum instability clades of CYP6DE subfamily isoforms in marked in color. **(B)** Minimum instability clades of CYP6DJ subfamily isoforms marked in color.

The instability analysis showed that cytochromes of CYP6DE and CYP6DJ subfamilies were divided into three and two minimum instability groups (MIGs), respectively (Figure 3). The first MIG from CYP6DE was integrated by cytochromes from the B and C phylogenetic subgroups with an instability score (IS) of 10.15, with four gene duplications and five losses. The other two MIGs from this family were integrated by cytochromes from phylogenetic subgroup A and Dpon-CYP6DE2 from phylogenetic subgroup B (Figure 3A), with an IS ≤ 2 and one and two loss events, respectively. The first MIG from the CYP6DJ included cytochromes from the phylogenetic subgroup X (Figure 3B), with IS = 4.57, two duplications and two loss events; the second MIG included the phylogenetic subgroup Y, with an IS < 1.43 and one loss event (Supplementary Table S4).

### 3.5 Molecular docking analysis of the cytochromes CYP6DE and CYP6DJ subfamilies

The structure assessment for cytochromes of CYP6DE and CYP6DJ subfamilies were 89.73%–94.14% of favored angles in the Ramachandran analysis, respectively. The ERRAT analysis of these isoforms showed a quality factor between 80.38-95.22 (Supplementary Table S2). All isoforms analyzed from the CYP6DE and CYP6DJ subfamilies presented favorable binding energy and Fe-C distance with enantiomers (+)- and (−)-*α*-pinene, (+)- and (−)-*β*-pinene, (+)-3-carene, *R*-(+)-limonene, and *β*-myrcene, except the Dpon-CYP6DJ1 whose interactions did not involve the catalytic site. None of tested monoterpenes interacted with the catalytic site (heme group) of Dpon-CYP6DJ1 (Supplementary Table S5). TM-scores among models of CYP6DE subfamily and CYP3A5 crystallized varied from 0.19 to 0.44, and among models CYP6DJ subfamily and CYP3A4 were above 0.2, and showing a high structural overlapping (Supplementary Figure S3).

#### 3.5.1 Phylogenetic subgroup A interactions

**FIGURE 4:**
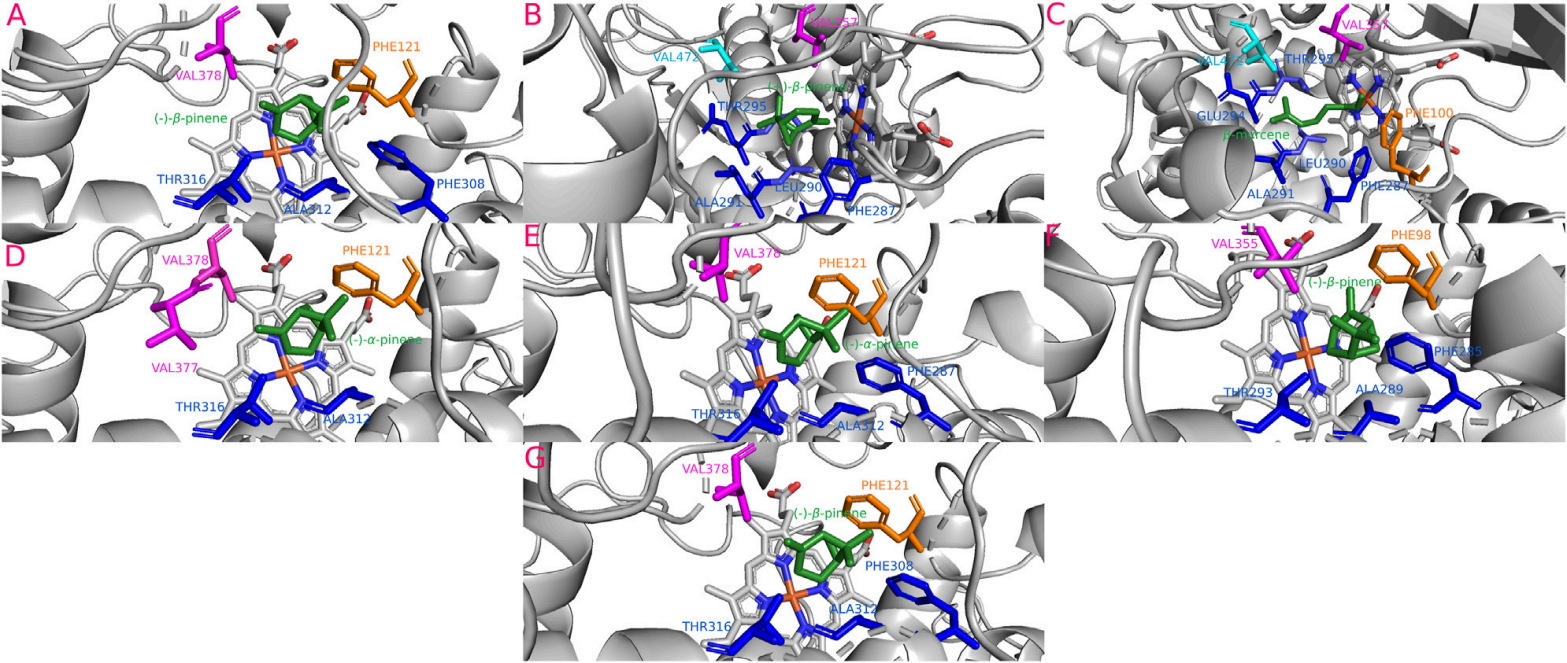
Most stable interactions in the molecular docking and between monoterpenes and cytochromes from phylogenetic subgroup A. Proteins are represented in gray, the catalytic site in blue-orange and most important interacting residues from SRSs with specific monoterpenes (green) in different color. **(A)** Dval-CYP6DE4 c. (−)-*β*-pinene, ΔG (Gibb’s free energy) = −25.79 kJ; **(B)** Drhi-CYP6DE4 c. (+)-*β*-pinene, ΔG = −23.06 kJ; **(C)** Dmex-CYP6DE1 c. *β*-Myrcene, ΔG = −25.03 kJ; **(D)** Dfro-CYP6DE2 c. (−)-*α*-pinene, ΔG = −25.07 kJ; **(E)** Dpon-CYP6DE4 c. (−)-*α*-pinene, ΔG = −25.95 kJ; **(F)** Darm-CYP6DE5 c. (−)-*β*-pinene, ΔG = −24.53 kJ; **(G)** Dfro-CYP6DE2 c. (−)-*β*-pinene, ΔG = −25.07 kJ.

Cytochromes from phylogenetic subgroup A formed the most stable conformations with (−)-*α*-pinene and (−)-*β*-pinene (−25.95 and −25.79 kJ/mol, respectively), except Drhi-CYP6DE4(A) and Dmex-CYP6DE(A)1 that showed the highest affinity with (+)-*β*-pinene (−23.06 kJ/mol) and *β-*myrcene (−25.03 kJ/mol) (Table 1). Dfro-CYP6DE(A)2 showed the same binding energy with both (−)-*α*-pinene and (−)-*β*-pinene (−25.07 kJ/mol). These interactions included the residues PHE (SRS1, SRS4), LEU (SRS4), ALA (SRS4), THR (SRS4), GLU (SRS4) and VAL (SRS5 and SRS6) (Figure 4). The Fe-C distances and predicted products were: 5.5 Å [Dmex-CYP6DE(A)1 c. *β-*myrcene] and (*R*)-(−)-ipsdienol; 4.7 Å [Dval-CYP6DE4(A), Dfro-CYP6DE(A)2 c. (−)-*β*-pinene] and (−)-*β*-pinene epoxide; 4.3 Å [Darm-CYP6DE5(A) c. (−)-*β*-pinene] and (−)-*β*-pinene epoxide; 2.8 Å [Dfro-CYP6DE(A)2, Dpon-CYP6DE4(A) c. (−)-*α*-pinene] and (−)-*trans*-verbenol (Table 1).

**TABLE 1:** Binding energy, closest carbon distance and predicted product from the most stable CYP-ligand interaction from each CYP6DE and CYP6DJ isoforms.

Phylogenetic sub-group	Isoform	Monoterpene	Binding energy (kJ/mol)	Oxygenated carbon	Distance (Å)	Predicted product
**A**	Dval-CYP6DE4	(−)-*β*-pinene	−25.79	C2	4.7	(−)-*β*-pinene epoxide
	Drhi-CYP6DE4	(+)-*β*-pinene	−23.06	Cmet	4.4	Myrtenol
	Dmex-CYP6DE1	*β*-myrcene	−25.03	C4	5.5	Ipsdienol
	Dfro-CYP6DE2	(−)-*α*-pinene	−25.07	C4	2.8	(−)-*trans*-verbenol
		(−)-*β*-pinene		C2	4.7	(−)-*β*-pinene epoxide
	Dpon-CYP6DE4	(−)-*α*-pinene	−25.95	C4	2.8	(−)-*trans*-verbenol
	Darm-CYP6DE5	(−)-*β*-pinene	−24.53	C2	4.3	(−)-*β*-pinene epoxide
**B**	Darm-CYP6DE6	(+)-*α*-pinene	−25.16	C4	4.1	(+)-*trans*-verbenol
	Dpon-CYP6DE2	(−)-*β*-pinene	−24.45	C2	5.2	(−)-*β*-pinene epoxido
	Drhi-CYP6DE3	(−)-*α*-pinene	−24.66	C4	3.9	(−)-*trans*-verbenol
**C**	Dpon-CYP6DE1	(+)-*α*-pinene	−24.82	C4	4.1	(+)-*trans*-verbenol
	Dpon-CYP6DE3	(+)-*β*-pinene	−23.19	C2	4.7	(+)-*β*-pinene epoxide
	Dmex-CYP6DE2	(+)-*α*-pinene	−25.07	C4	4	(+)-*trans*-verbenol
	Dfro-CYP6DE1	(−)-*β*-pinene	−24.66	C2	4.4	(−)-*β*-pinene epoxide
	Dadj-CYP6DE2	(+)-*α*-pinene	−25.16	C4	4.1	(+)-*trans*-verbenol
	Dadj-CYP6DE1	(−)-*β*-pinene	−24.82	C2	5.5	(−)-*β*-pinene epoxide
	Dadj-CYP6DE3	(+)-*β*-pinene	−23.61	Cmet	3.5	Myrtenol
	Drhi-CYP6DE1	(+)-*β*-pinene	−23.27	C2	4.8	(+)-*β*-pinene epoxide
	Dval-CYP6DE1	(+)-*β*-pinene	−23.27	Cmet	2.6	Myrtenol
**X**	Dadj-CYP6DJ2-1	(+)-*β*-pinene	−27.92	C2	6.1	(+)-*β*-pinene epoxide
	Dadj-CYP6DJ2-2	(−)-*α*-pinene	−26.87	C4	3.7	(−)-*trans*-verbenol
	Dpon-CYP6DJ2	(−)-*α*-pinene	−27.13	C4	3.7	(−)-*trans*-verbenol
	Drhi-CYP6DJ2	(−)-*α*-pinene	−27.04	C4	3.7	(−)-*trans*-verbenol
	Dfro-CYP6DJ2-1	(−)-*α*-pinene, (+)-*β*-pinene	−26.83	Cmet	4.8	Myrtenol (+)-*β*-pinene epoxide
				C2	5.2	
	Dfro-CYP6DJ2-2	(−)-*α*-pinene	−26.46	Cmet	4.9	Myrtenol
	Darm-CYP6DJ2	(−)-*β*-pinene	−26.04	C2	3.8	(−)-*β*-pinene epoxide
	Dval-CYP6DJ2	(+)-*β*-pinene	−26.79	C2	5.1	(+)-*β*-pinene epoxide
**Y**	Dadj-CYP6DJ1	(+)-*α*-pinene	−24.20	Cmet	4.9	Myrtenol
	Dmex-CYP6DJ1	(−)-*α*-pinene	−24.47	Cmet	3	Myrtenol
	Dfro-CYP6DJ1	(+)-*β*-pinene	−24.15	C2	4.3	(+)-*β*-pinene epoxide
	Dpon-CYP6DJ1	N/A	N/A	N/A	N/A	N/A
	Drhi-CYP6DJ1	(+)-*β*-pinene	−26.37	C2	5.4	(+)-*β*-pinene epoxide
	Dval-CYP6DJ1	(+)-*α*-pinene, (+)-*β*-pinene	−27.08	C4	4.0	(+)-*trans*-verbenol
				C2	5.4	(+)-*β*-pinene epoxide

#### 3.5.2 Phylogenetic subgroup B interactions

**FIGURE 5:**
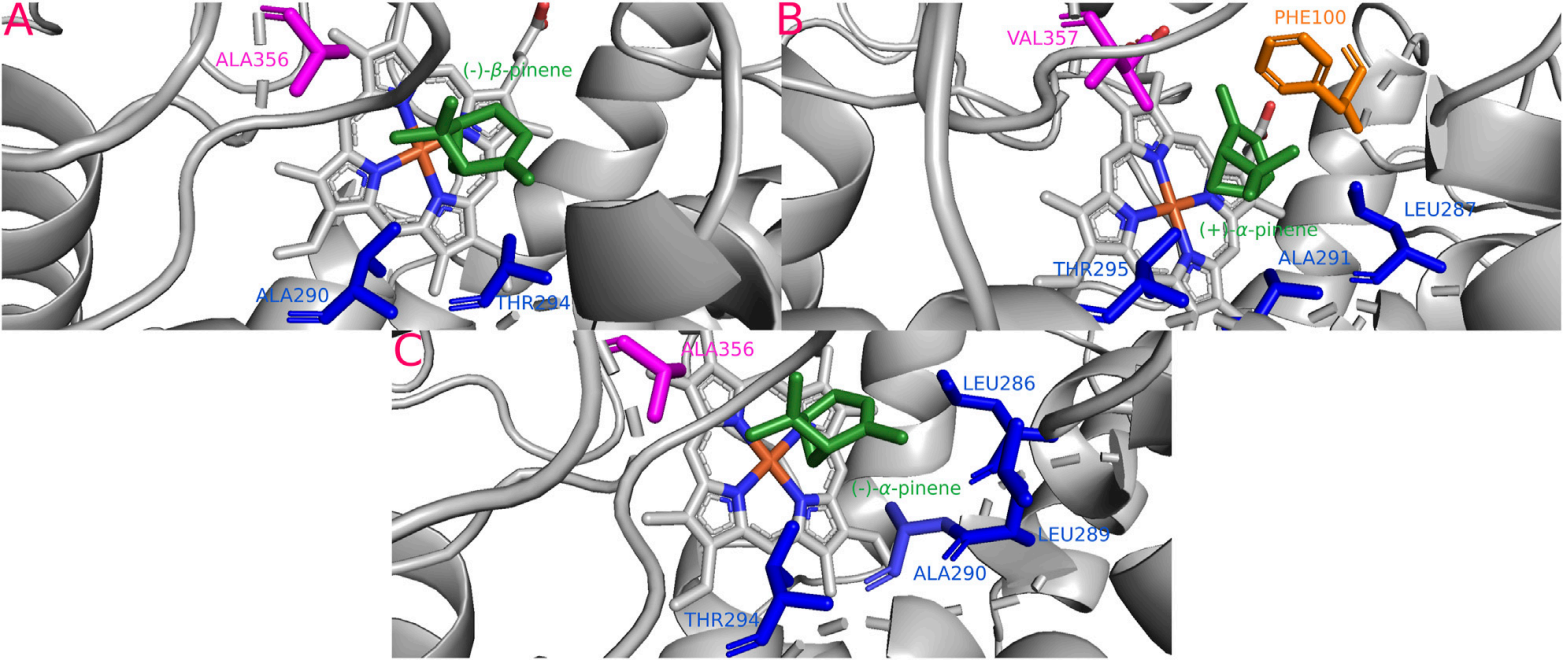
Most stable interactions in the molecular docking between monoterpenes and cytochromes from phylogenetic subgroup B. Proteins are represented in gray, the catalytic site in blue and most important interacting residues from SRSs with specific monoterpenes (green) in different color. **(A)** Dpon-CYP6DE2 c. (−)-*β*-pinene, ΔG (Gibb’s free energy) = −24.45 kJ; **(B)** Darm-CYP6DE6 c. (+)-*α*-pinene, ΔG = −25.16 kJ; **(C)** Drhi-CYP6DE3 c. (+)-*α*-pinene, ΔG = −24.66 kJ.

The isoform Darm-CYP6DE6(B) showed the highest affinity with (+)-*α*-pinene (−25.16 kJ/mol), Dpon-CYP6DE2(B) with (−)-*β*-pinene (−24.45 kJ/mol), and Drhi-CYP6DE3(B) with (−)-*α*-pinene (−24.66 kJ/mol) (Table 1). These interactions included the residues PHE (SRS1), LEU (SRS4), ALA (SRS4, SRS5), THR (SRS4), and VAL (SRS5) (Figure 5). The Fe-C distances and derived products of these interactions were: 5.2 Å [Dpon-CYP6DE2(B) c. (−)-*β*-pinene] and (−)-*β*-pinene epoxide 4.1 Å Darm-CYP6DE6(B) c. (+)-*α*-pinene] and (+)-*trans*-verbenol; 3.9 Å [Drhi-CYP6DE3(B) c. (−)-*α*-pinene] and (−)-*trans*-verbenol (Table 1).

#### 3.5.3 Phylogenetic subgroup C interactions

**FIGURE 6:**
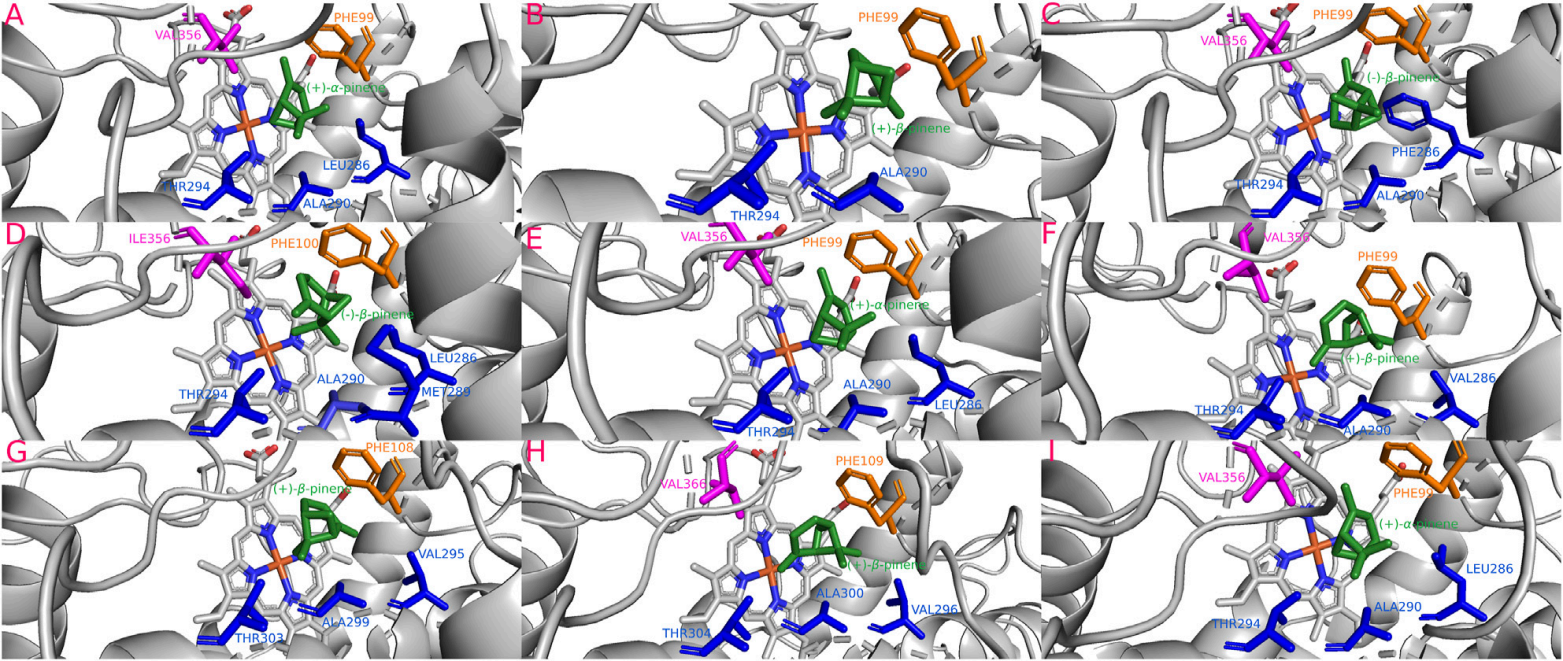
Most stable interactions in the molecular docking between monoterpenes and cytochromes from phylogenetic subgroup C. Proteins are represented in gray, the catalytic site in blue-orange and most important interacting residues from SRSs with specific monoterpenes (green) in different color. **(A)** Dpon-CYP6DE1 c. (+)-*α*-pinene, ΔG (Gibb’s free energy) = −24.82 kJ; **(B)** Dpon-CYP6DE3 c. (+)-*β*-pinene, ΔG = −23.19 kJ; **(C)** Dfro-CYP6DE1 c. (−)-*β*-pinene, ΔG = −24.66 kJ; **(D)** Dadj-CYP6DE1 c. (−)-*β*-pinene, ΔG = −24.82 kJ; **(E)** Dadj-CYP6DE2 c. (+)-*α*-pinene, ΔG = −25.16 kJ; **(F)** Dadj-CYP6DE3 c. (+)-*β*-pinene, ΔG = −23.61 kJ; **(G)** Drhi-CYP6DE1 c. (+)-*β*-pinene, ΔG = −23.27 kJ; **(H)** Dval-CYP6DE1 c. (+)-*β*-pinene, ΔG = −23.27 kJ; **(I)** Dmex-CYP6DE2 c. (+)-*α*-pinene, ΔG = −25.07 kJ.

The isoforms Dpon-CYP6DE3(C) (−23.19 kJ/mol), Dadj-CYP6DE(C)3 (−23.61 kJ/mol), Drhi-CYP6DE1(C) (−23.27 kJ/mol), and Dval-CYP6DE1(C) (−23.27 kJ/mol) showed a more stable complex with (+)-*β*-pinene, although Dfro-CYP6DE(C)1 (−24.66 kJ/mol) and Dadj-CYP6DE(C)1 (−24.82 kJ/mol) also formed a complex with (−)-*β*-pinene. Dpon-CYP6DE1(C) (−24.82 kJ/mol), Dmex-CYP6DE(C)2 (−25.07 kJ/mol), and Dadj-CYP6DE(C)2 (−25.16 kJ/mol) had the highest affinity with (+)-*α*-pinene (Table 1). These interactions included the residues PHE (SRS1), LEU (SRS4), ALA (SRS4), THR (SRS4), VAL (SRS4, SRS5), ILE (SRS5), and MET (SRS4). The last two residues were only present in the interaction of Dadj-CYP6DE(C)1 with (−)-*β*-pinene (Figure 6). The Fe-C distances and predicted products were: 5.5 Å [Dadj-CYP6DE(C)1 c. (−)-*β*-pinene] and (−)-*β*-pinene epoxide; 4.8 Å [Drhi-CYP6DE(C)1 c. (+)-*β*-pinene] and (+)-*β*-pinene epoxide; 4.7 Å [Dpon-CYP6DE(C)3 c. (+)-*β*-pinene] and (+)-*β*-pinene epoxide; 4.4 Å [Dfro-CYP6DE(C)1 c. (−)-*β*-pinene] and (−)-*β*-pinene epoxide; 4.1 Å Dpon-CYP6DE1(C), Dmex-CYP6DE(C)2, and Dadj-CYP6DE(C)2 c. (+)-*α*-pinene] and (+)-*trans*-verbenol in three cases; 3.5 Å (Dadj-CYP6DE(C)3 and 2.6 Å [Dval-CYP6DE(C)1 both c. (+)-*β*-pinene and myrtenol (Table 1).

#### 3.5.4 Phylogenetic subgroup X interactions

**FIGURE 7:**
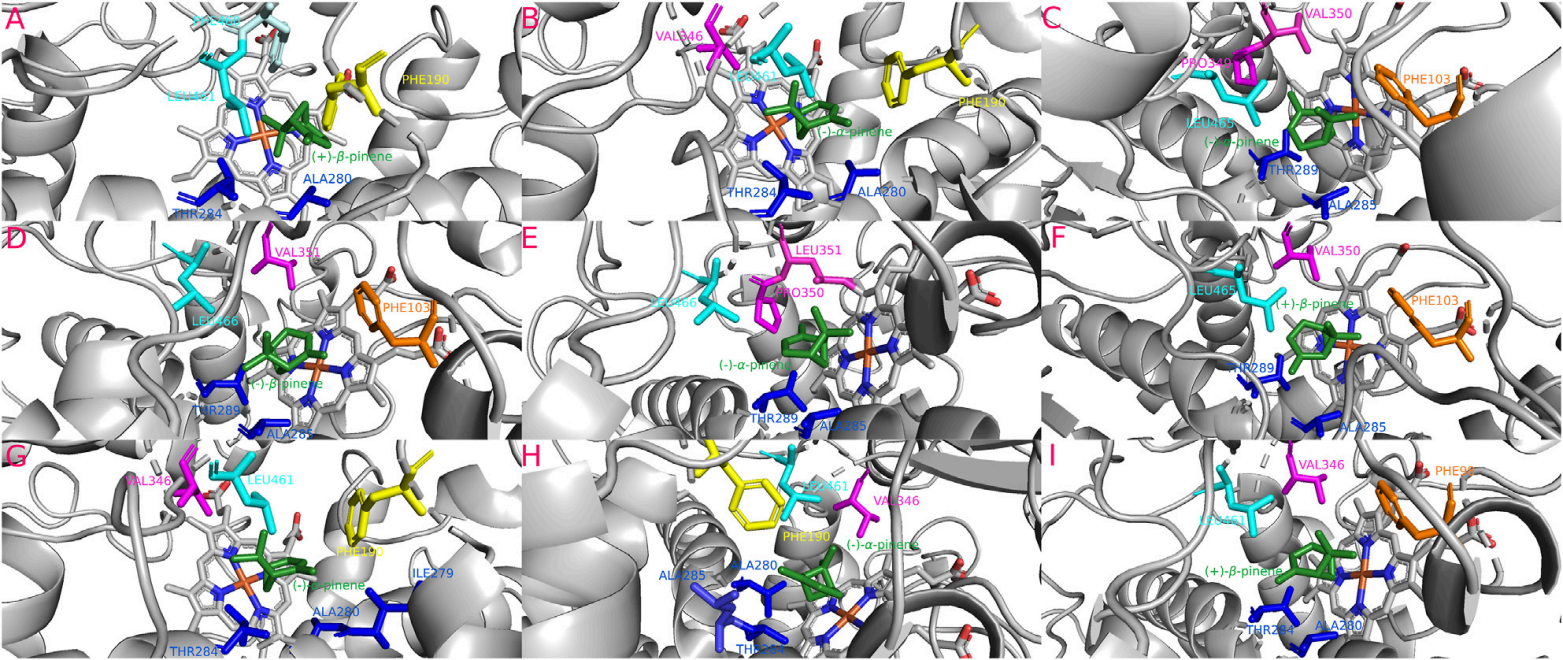
Most stable interactions in the molecular docking between monoterpenes and cytochromes from phylogenetic subgroup X. Proteins are represented in gray, the catalytic site in blue-orange and most important interacting residues from SRSs with specific monoterpenes (green) in different color. **(A)** Dadj-CYP6DJ2-1 c. (+)-*β*-pinene, ΔG (Gibb’s free energy) = −27.92 kJ; **(B)** Dadj-CYP6DJ2-2 c. (−)-*α*-pinene, ΔG = −26.87 kJ; **(C)** Dfro-CYP6DJ2-1 c. (−)-*α*-pinene, ΔG = −26.83 kJ; **(D)** Darm-CYP6DJ2 c. (−)-*β*-pinene, ΔG = −26.04 kJ; **(E)** Dfro-CYP6DJ2-2 c. (−)-*α*-pinene, ΔG = −26.46 kJ; **(F)** Dfro-CYP6DJ2-1 c. (+)-*β*-pinene, ΔG = −26.83 kJ; **(G)** Dpon-CYP6DJ2 c. (−)-*α*-pinene, ΔG = −27.13 kJ; **(H)** Drhi-CYP6DJ2 c. (−)-*α*-pinene, ΔG = −27.04 kJ; **(I)** Dval-CYP6DJ2 c. (+)-*β*-pinene, ΔG = −26.79 kJ.

The isoforms Dadj-CYP6DJ(X)2-2 (−26.87 kJ/mol), Dpon-CYP6DJ2(X) (−27.13 kJ/mol), Drhi-CYP6DJ2(X) (−27.04 kJ/mol), Dfro-CYP6DJ(X)2-1 (−26.83 kJ/mol), and Dfro-CYP6DJ(X)2-2 (−26.46 kJ/mol) showed the most stable interaction with (−)-*α*-pinene; Dadj-CYP6DJ(X)2-1 (−27.92 kJ/mol), and Dval-CYP6DJ2(X) (−26.79 kJ/mol) with (+)-*β*-pinene, and Darm-CYP6DJ2(X) with (−)-*β*-pinene (−26.04 kJ/mol) (Table 1). Only Dfro-CYP6DJ(X)2-1 presented equal binding energy with both (−)-*α*-pinene and (+)-*β*-pinene (−26.83 kJ/mol). These interactions included the residues PHE (SRS1, SRS3, SRS6), ALA (SRS4), THR (SRS4), ILE (SRS4), PRO (SRS5) and LEU (SRS5, SRS6). The PRO residue was only present in interactions of Dfro-CYP6DJ(X)2-1, and Dfro-CYP6DJ(X)2-2. This shows that interactions were more frequent with SRS3, SRS5 and SRS6 from cytochromes of CYP6DJ subfamily than with those from the CYP6DE subfamily (Figure 7). The Fe-C distances and the predicted products were: 6.1 Å [Dadj-CYP6DJ(X)2-1 c. (+)-*β*-pinene] and (+)-*β*-pinene epoxide; 5.2 Å [Dfro-CYP6DJ(X)2-1 c. (+)-*β*-pinene] and (+)-*β*-pinene epoxide; 5.1 Å [Dval-CYP6DJ2(X) c. (+)-*β*-pinene] and (+)-*β*-pinene epoxide; 4.8 Å and 4.9 Å [Dfro-CYP6DJ(X)2-1, Dfro-CYP6DJ(X)2-2 c. (−)-*α*-pinene] and myrtenol; 3.8 Å Darm-CYP6DJ2(X) c. (−)-*β*-pinene] and (−)-*β*-pinene epoxide; 3.7 Å [Dadj-CYP6DJ(X)2-2, Dpon-CYP6DJ2(X), and Drhi-CYP6DJ2(X) c. (−)-*α*-pinene] and (−)-*trans*-verbenol in the three cases (Table 1).

#### 3.5.5 Phylogenetic subgroup Y interactions

**FIGURE 8:**
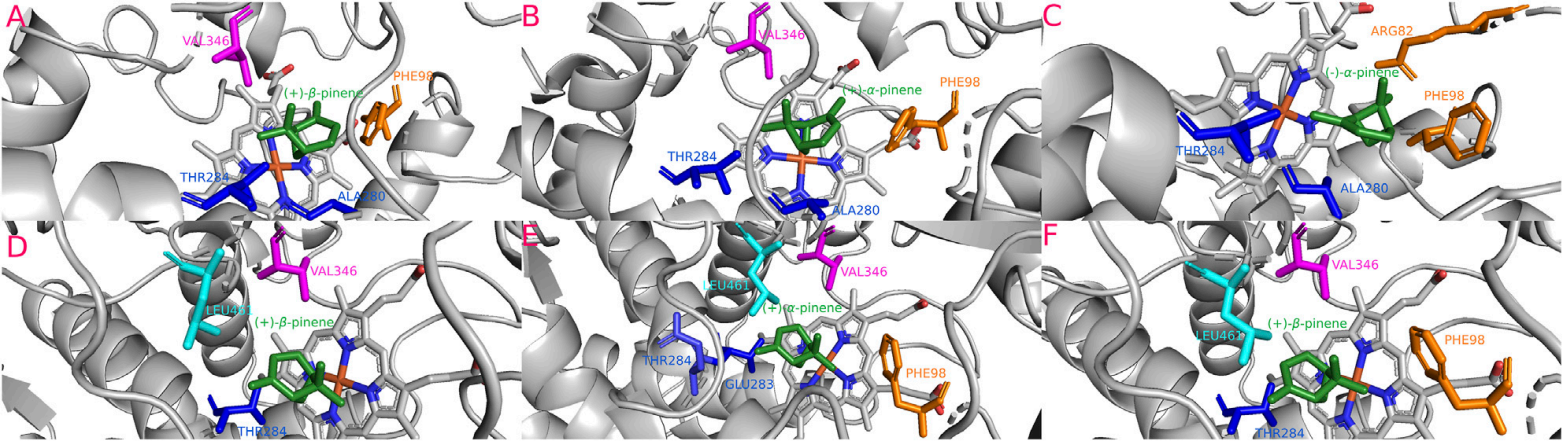
Most stable interactions in the molecular docking between monoterpenes and cytochromes from phylogenetic subgroup Y. Proteins are represented in gray, the catalytic site in blue-orange and most important interacting residues from SRSs with specific monoterpenes (green) in different color. **(A)** Dfro-CYP6DJ1 c. (+)-*β*-pinene, ΔG (Gibb’s free energy) = −24.15 kJ; **(B)** Dadj-CYP6DJ1 c. (+)-*α*-pinene, ΔG = −24.2 kJ; **(C)** Dmex-CYP6DJ1 c. (−)-*α*-pinene, ΔG = −24.47 kJ; **(D)** Drhi-CYP6DJ1 c. (+)-*β*-pinene, ΔG = −26.37 kJ; **(E)** Dval-CYP6DJ1 c. (+)-*α*-pinene, ΔG = −27.08 kJ; **(F)** Dval-CYP6DJ1 c. (+)-*β*-pinene, ΔG = −27.08 kJ.

The isoforms Dadj-CYP6DJ(Y)1 (−24.2 kJ/mol), Dmex-CYP6DJ(Y)1 (−24.47 kJ/mol), and Dval-CYP6DJ1(Y) (−27.08 kJ/mol) had the most stable interaction with (+)- and (−)-*α*-pinene and; Dval-CYP6DJ1(Y) (−27.08 kJ/mol) Dfro-CYP6DJ(Y)1 (−24.15 kJ/mol) and Drhi-CYP6DJ1(Y) (−26.37 kJ/mol) with (+)-*β*-pinene (Table 1). Dpon-CYP6DJ1(Y) interacted with both *α-* and *β*-pinene enantiomers but did not include the heme-group. Interactions included the residues ARG (SRS1), PHE (SRS1), ALA (SRS4), THR (SRS4), GLU (SRS4), VAL (SRS5), and LEU (SRS6). The LEU residue was only present in interactions of Drhi-CYP6DJ1(Y) and Dval-CYP6DJ1(Y), while ARG was only in interactions of Dmex-CYP6DJ(Y)1, which lacked the VAL residue. The GLU residue was only present in the interaction of Dval-CYP6DJ1(Y) with (+)-*α*-pinene, while Drhi-CYP6DJ1(Y) interacted with the same terpene but the PHE residue was absent (Figure 8). The Fe-C distances and predicted products were: 5.4 Å [Drhi-CYP6DJ1(Y), Dval-CYP6DJ1(Y) c. (+)-*β*-pinene] and (+)-*β*-pinene epoxide in both cases; 4.9 Å [Dadj-CYP6DJ(Y)1 c. (+)-*α*-pinene] and myrtenol; 4.3 Å [Dfro-CYP6DJ(Y)1 c. (+)-*β*-pinene] and (+)-*β*-pinene epoxide; 4.0 Å [Dval-CYP6DJ1(Y) c. (+)-*α*-pinene] and (+)-*trans*-verbenol; 3.0 Å [Dmex-CYP6DJ(Y)1 c. (−)-*α*-pinene] and myrtenol (Table 1).

### 3.6 Functional divergence analysis

Statistically significant type-I functional divergence was found between the phylogenetic subgroups BC and A (θ_I_ = 0.999,428 ± 0.131,040 > 0, *p < 0.05) (Supplementary Table S6). A total of 182 amino acid residues were identified as critical sites (CAASs), located in regions 160-262 from SRS2 and SRS3, and 264-344 from SRS4 and K-helix with a posterior probability (Qk) > 0.7, specifically residues 183-192 spotted in SRS2, 217-225 in SRS3, 273-291 in SRS4, and 341-344 on K-helix (Supplementary Figure S1).

Type-II functional divergence was also statistically significant (θ_II_ = 0.247,328 ± 0.071711> 0, *p < 0.05). A total of 54 CAASs were identified, including the positions 159 to 343 (Qk > 0.7), specifically residues 184,190,191 were in SRS2; 219 and 222-224 in SRS3; 272, 276, 280 in SRS4, and only 343 in the K-helix motif; the rest (43 CAASs) were not located in some domain or motif (Supplementary Figure S1). The CAASs changes in both types of functional divergence were mainly of polar vs non-polar residues (Supplementary Table S6).

There was no statistical significance in Type-I functional divergence between subgroups X and Y from the CYP6DJ subfamily (θ_I_ = 0.294,284 ± 0.09737 > 0, *p < 0.05). Only four residues (201, 228, 370 and 467) were identified as CAASs (Qk > 0.7), and they did not fall in some domain or motif (Supplementary Figure S2). Type-II divergence between same phylogenetic subgroups from the CYP6DJ subfamily was not statistically significant (θ_II_ < 0.001584 ± 0.038377, *p* < 0.05). The variation in CAASs corresponded mainly to exchanges between polar vs non-polar residues (Supplementary Table S6).

## 4 Discussion

### 4.1 Phylogenetic analysis of the CYP6 family members

The inferred phylogeny shows orthology patterns among different phylogenetic subgroups within CYP6DE and CYP6DJ subfamilies from *Dendroctonus*-species (Figure 1). In particular, the mapping of phylogenetic subgroups in the *Dendroctonus*-phylogeny suggests that gain and loss events occurred during the diversification of these bark beetles (Figure 2).

The loss or gain of isoforms from the phylogenetic subgroups align with the birth-death model of evolution proposed for the P450 superfamily platform (Feyereisen, 2011), which results from retroposition, duplication or deletion events, as well as mutation, genetic drift, and selection. In the case of *Dendroctonus*-species, the retention, gain or loss of certain phylogenetic subgroups could be thought is associated to chromosomic changes, because these bark beetles have different chromosome numbers (*D. adjunctus* 6AA + Xyp, *D. frontalis* 7AA+ Xyp, *D. mexicanus* (5AA + Xyp, *D. ponderosae* 11AA+ neoXY, *D. rhizophagus* 13AA+ Xyp, and *D. valens* 13AA+ Xyp) (Lanier, 1981; Zúñiga et al., 2002a; Zúñiga et al., 2002b; Bracewell et al., 2017). Yet, although the gain of phylogenetic subgroups C and Y, relative to the basal species *D. armandi*, may increase the molecular plasticity of species, their loss (e.g., A, B, and X) in several species apparently has no negative effect on their fitness, since they face the same subcortical environment that is heterogeneous and changing in space and time. This confirms that physiological functions are not restricted to specific group or subfamilies of the P450 cytochromes (Feyereisen, 2012).

### 4.2 Analysis of full-length cytochrome CYP6DE and CYP6DJ

The *in silico* analyses of CYP6DE and CYP6DJ subfamilies indicated that the cytochromes are anchored to the endoplasmic reticulum membrane, as reported for other families such as CYP4, CYP6 and CYP9s of the *Dendroctonus* species (Cano-Ramírez et al., 2013; López et al., 2013; Dai et al., 2014).

Isoforms from different subfamilies have low identities, yet they share a common structural fold with well-defined secondary structure elements, motifs, and domains (Sirim et al., 2010). Multiple alignments of cytochromes from CYP6DE and CYP6DJ subfamilies revealed highly conserved sites, such as the K-helix (EXXR) and PERF (PXXF) that are fundamental for cytochrome structure and stability, as well as the heme-binding site (FGXGPRXCXG) which constitutes the catalytic site and the signature of cytochrome P450 (Feyereisen, 2011; Feyereisen, 2012). The SRS sequences from CYP6DE and CYP6DJ subfamilies showed differences between them, as was reported for other isoforms of the CYP6 family from *Dendroctonus* spp. and other insect species (Cano-Ramírez et al., 2013; López et al., 2013; Li et al., 2014; Zhang et al., 2019; Liu et al., 2022; Liu and Chen, 2022).

Among the analyzed subfamilies, results have shown that SRSs 1, 2, 3, and 6 are the most variable, which agrees with the observation in CYP6AE from *Helicoverpa armigera* (Shi et al., 2020). The SRSs vary as a result of mutations that produce changes in protein folding, thereby modifying the isoforms’ specificity towards the substrate (Schuler and Berenbaum, 2013). These mutations could lead to changes that increase the ability of isoforms to metabolize one type of substrate, as has been demonstrated in isoforms of the families CYP2 and CYP3 from rabbit, mouse, and human (Zawaira et al., 2008). In the case of SRS4 and SRS5, they are also variable, but present more conserved sites than SRSs 1, 2, 3, and 6 (Supplementary Figures S1, S2). These changes determine the correct folding of the catalytic site of these enzymes (Schuler and Berenbaum, 2013).

The SRSs of CYPs from herbivorous insects, including bark beetles, are under selective pressure and favor the recognition of a wide number of substrates due to the interaction of insects with a plethora of secondary metabolites in their host trees (Feyereisen, 2011; Schuler and Berenbaum, 2013; Li et al., 2014; Calla, 2021). In the case of bark beetles, they should metabolize different monoterpenes and diterpenes, many of them highly toxic (*e.g*., pinenes, limonene, 3-carene, myrcene, terpinolene, and phellandrene) and yielding products which synergize the action of pheromonal compounds or function well as pheromones (*e.g., trans*-verbenol, ipsdienol, and myrtenol) that favor massive attacks of conspecifics to overcome host resistance (Chiu et al., 2017; Blomquist et al., 2021).

### 4.3 Molecular docking

#### 4.3.1 Residues involved in the CYP6DE and CYP6DJ molecular interactions

Our findings showed that the molecular interactions between cytochromes of the CYP6DE and CYP6DJ subfamilies and monoterpenes included the catalytic site (heme group) and several SRSs, with SRS1, SRS4 and SRS5 being the most frequent. In the five subgroups, PHE, LEU, ALA, and VAL were the amino acids present in all receptor-ligand interactions (Figures 4–8). These non-polar amino acids stabilize the catalytic site and determine the substrates that can be metabolized by different isoforms. Specifically, PHE and VAL residues constitute an aromatic network that forms the catalytic pocket, which stabilizes bond strength with the substrate (Chen et al., 2002; Baudry et al., 2003; Li et al., 2004; Shi et al., 2020). Both residues are present many times in a single conformation, as can be observed in the CYP6DJ2 isoforms of *Dendroctonus* species analyzed, and whose binding energy is higher with (−)-*α*-pinene and (−)-*β*-pinene compared to the CYP6DE isoforms (Table 1). Interestingly, the LEU286 residue participates in all interactions of Dpon-CYP6DE1(C) and Drhi-CYP6DE3(B) with different substrates, which influences the catalytic site conformation of these isoforms, as has been documented with CYP6AE in *H. armigera* (Shi et al., 2020).

The results also showed that the PRO residue from SRS5 is present in all interactions with *β*-myrcene (Supplementary Table S5). It has been reported that this residue interacts with other aromatic residues, such as PHE, thereby favoring the molecular interaction (Bhattacharyya and Chakrabarti, 2003). The presence of PRO and PHE residues in all interactions with *β*-myrcene, suggests that the interaction of both residues with this terpene is conserved, except in Dpon-CYP6DJ1(Y), despite myrcene is a toxic compound and abundant in mature pine tree colonized by *D. ponderosae* (Smith, 2000; Chiu et al., 2017).

The cytochrome-monoterpene conformations showed stereoselectivity with respect to *α*-pinene and *β*-pinene enantiomers. Dpon-CYP6DE1(C) and 3(C) isoforms form the most stable complexes with (+) enantiomers, whereas Dpon-CYP6DE2(B) with (−) enantiomers of both terpenes (Table 1). The main difference between Dpon-CYP6DE1(C), 3(C) and 2(B) interactions is the absence of the PHE99 residue in this last, suggesting that other residues outside the SRSs might influence its conformational stability. On the other hand, the orthologs Drhi-CYP6DE1(C) and Dval-CYP6DE1(C) constitute the most stable conformations with the same enantiomer, (+)-*β*-pinene, but with different distance values in Dval-CYP6DE1(C) due to the presence of the VAL366 residue which stabilizes the substrate binding, as observed in CYP6B1v1 and CYP6B1 of *Papilio polyxenes*, and CYP6B8 of *H. zea* (Chen et al., 2002; Li et al., 2004).

#### 4.3.2 Phylogenetic subgroups A, B, and C interactions

Our mapping and phylogenetic inference results showed that subgroups A and B from CYP6DE are ancestral, as suggested by their presence in the basal species *D. armandi* in the *Dendroctonus*-phylogeny (Figure 2). The retention of subgroup A in almost all species suggests that their functional activity is fundamental to *Dendroctonus*-species. This is not the case for the phylogenetic subgroup B, a paralog of subgroup A also present in *D. armandi*, because this subgroup was later lost in the *D. frontalis* complex species studied (*D. adjunctus*, *D. mexicanus*, and *D. frontalis*) and *D. valens*. The evolutionary history of subgroup C is different because it emerged after the segregation of *D. armandi* and was retained during the diversification of *Dendroctonus* species. Apparently, the loss of subgroup B in the *D. frontalis* complex species was offset by the gain of subgroup C (Figure 2).

Docking analyses showed that phylogenetic subgroups A, B, and C have molecular interactions mainly with the same monoterpenes, although there are specific particularities in each subgroup. For example, the most stable conformations of cytochromes from subgroup A are mainly associated with enantiomers metabolism from *α*- and *β*-pinene, except Dmex-CYP6DE(A)1 c. *β*-myrcene (Table 1). Experimental evidence has shown that the silencing of Darm-CYP6DE5(A) resulted in an increase adult insect mortality of *D. armandi,* after exposure to these terpenoid compounds (Liu et al., 2022). The main product of (−)-*β*-pinene hydroxylation, myrtenol, is not known as a pheromone in this bark beetle (Chen et al., 2015), as well as in *D. rhizophagus* (Cano-Ramírez et al., 2012), *D. valens* (Shi and Sun, 2010), and *D. frontalis* (Sullivan, 2016). Our findings suggest that the biological role of these cytochromes in these species is directly related to the detoxification process of *β*-pinene enantiomers.

In the case of Dpon-CYP&DE4(A), the most stable conformation was with (−)-*α*-pinene, because of the shorter Fe-C distance compared to other monoterpenes. This suggests that the transformation of (−)-*α*-pinene to (−)-*trans*-verbenol is highly specific, due perhaps to the importance that (−)-*trans*-verbenol has as an aggregation pheromone in *D. ponderosae* (Chiu and Bohlmann, 2022), independent of whether (−)-*trans*-verbenol is the result of direct hydroxylation of (−)-*α*-pinene in the tree oleoresin or from the verbenyl esters accumulated in the early stages of development and their subsequent release by females in the adult stage (Chiu et al., 2018).

The Dmex-CYP6DE(A)1 cytochrome showed the highest affinity towards *β*-myrcene, which agrees with the production of (*R*)-(−)-ipsdienol in *D. mexicanus*. Behavioral and electrophysiology studies have demonstrated that this species produces (*R*)-(−)-ipsdienol which apparently acts as an aggregation pheromone (Cano-Ramírez pers. comm.). In bark beetles of the genus *Ips*, (*R*)-(−)-ipsdienol is produced by myrcene hydroxylation, which is synthetized *de novo* and hydroxylated by CYP9T1, 2, 3 (Sandstrom et al., 2006; Sandstrom et al., 2008). *Dendroctonus*-bark beetles do not have genes from the CYP9T subfamily, hence future experimental studies should be performed to test whether Dmex-CYP6DE(A)1 is able to hydroxylate myrcene to ipsdienol.

With respect to subgroup B, our findings showed different interactions with the monoterpenes analyzed. The retention of Darm-CYP6DE6(B) isoform in *D. armandi* has reinforced the detoxification capacity of the paralogous Darm-CYP6DE5(A) isoform, as the former showed a very stable interaction with (+)-*α*-pinene, where the latter presented less specific interactions with monoterpenes, despite showing preference for (−)-*β*-pinene. An interesting case are the results of interactions between Dpon-CYP6DE2(B) from *D. ponderosae* with different monoterpenes, which suggested that this isoform can interact with all tested compounds. Nevertheless, experimental evidence with this enzyme did not show functional activity on different monoterpenes and diterpenes (Chiu et al., 2019a). The authors proposed that the inactivity could be due to the lack of knowledge about optimal conditions (e.g., pH, temperature, concentration) to experimentally hydroxylate the different terpenes. Another explanation could be the presence of unspecific residues involved in the interaction between cytochrome P450-reductase (CPR) and the cytochrome Dpon-CYP6DE2(B), as has been documented with GLY 217 and THR 402 residues of cytochromes CYP6AS7 and CYP6AS8 from the orchid bee *Euglossa dilemma* (Darragh et al., 2021). Results with Drhi-CYP6DE3(B) also showed a very stable interaction with (−)-*α*-pinene, whose hydroxylation produces mainly (−)-*trans*-verbenol. This could be similar to what occurs in *D. ponderosae*, as it has been suggested that (−)-*trans*-verbenol is the sex pheromone of *D. rhizophagus* (Cano-Ramírez et al., 2012).

Lastly, molecular interactions from phylogenetic subgroup C cytochromes showed that their acquisition is a reinforcement or an offset of phylogenetic subgroups A and B cytochromes. The Drhi-CYP6DE1(C) has a functional activity in *D. rhizophagus* that enforces subgroup A isoforms, because it has the same preferential substrate, (+)-*β*-pinene, whose hydroxylation produces an intermediary epoxide of *β*-pinene which yields myrtanal, myrtenol or perillyl alcohol. The findings with Dpon-CYP6DE1(C) and Dpon-CYP6DE3(C) isoforms from *D. ponderosae* are similar to those found in subgroup B, because both can hydroxylate all assayed monoterpenes. These results agree with the experimental assays performed with these isoforms (Nadeau et al., 2017; Chiu et al., 2019a).

Based on molecular interactions and the shorter Fe-C distances toward C4, the preferred substrate of Dpon-CYP6DE1(C) is (+)-*α*-pinene, whereas Dpon-CYP6DE3(C) prefers (+)-*β*-pinene whose hydroxylation produces (+)-*trans*-verbenol and a *β*-pinene epoxide, respectively. As mentioned above, if the experimental results with the Dpon-CYP6DE2(B) isoform are correct (Chiu et al., 2019a), despite our predictions, then the loss of function in subgroup B would be a pseudogenization, and therefore subgroup C cytochromes would offset this loss. In *D. valens*, the presence of Dval-CYP6DE1(C) is a compensation to the subgroup A isoform, because subgroup B is absent. The isoforms from subgroups A and C prefer *β*-pinene enantiomers as substrates, whose hydroxylation yields myrtenol, myrtanal and perillyl alcohol as end products. Myrtenol has been reported in *D. valens* as a synergist compound of kairomones and pheromones attractive of this species (Zhang and Sun, 2006; Shi and Sun, 2010).

The isoforms profit from subgroup C in the *D. frontalis* complex species which is an offset to the loss of subgroup B isoforms, but in *D. adjunctus* to subgroup A isoforms. Our findings with this species showed that the three isoforms from subgroup C (Dadj-CYP6DE(C)1,2,3 form the most stable complexes with enantiomers (+)-*α*-pinene and (+)- and (−)-*β*-pinene, whose hydroxylation produces (+)-*trans*-verbenol, myrtenol, and a *β*-pinene-epoxide. Advanced studies indicated that myrcene is an important kairomone for this species, but our findings showed that the interaction of isoforms Dadj-CYP6DE(C)1,2,3 with myrcene is not stable (Supplementary Table S5). Likewise, the findings showed that Dfro-CYP6DE(C)1 and Dmex-CYP6DE(C)2 interacted with all assayed monoterpenes but prefer (−)-*β*-pinene and (+)-*α*-pinene, respectively. The first is not involved in the chemical ecology of *D. frontalis* (Chen et al., 2002), while the second acts as a kairomone in *D. mexicanus* (Cano-Ramírez pers. comm.).

A point that needs to be highlighted about these two bark beetles is that while subgroup C in *D. frontalis* reinforces the functional activity of the ancestral subgroup A, in *D. mexicanus* there is a differential preference with respect to the hydroxylated monoterpene, because the isoform of subgroup C hydroxylates (+)-*α*-pinene to produce (+)-*trans*-verbenol and the isoform from subgroup A hydroxylates myrcene producing ipsdienol, a pheromone of this species.

#### 4.3.3 Phylogenetic subgroups X and Y interactions

Our findings showed that isoforms from phylogenetic subgroup X had the most stable interactions with enantiomers (−)-*α*-pinene and (+)- and (−)-*β*-pinene, whose main products were (−)-*trans*-verbenol and myrtenol (Table 1). The latter is produced from the hydrolysis of (−)-*α*-pinene only by Dfro-CYP6DJ(X)2-1and 2-2. These findings showed that isoforms from subgroup X are involved mainly in the detoxification process of monoterpenes, as reported in other studies (Dai et al., 2015; Obregón-Molina et al., 2015; Sarabia et al., 2019; Liu and Chen, 2022; Torres-Banda et al., 2022), independently that (−)-*trans*-verbenol can be used by some of these species as sexual (*D. rhizophagus*) or aggregation (*D. ponderosae*) pheromone (Borden et al., 1987; Cano-Ramírez et al., 2012) and myrtenol as a synergistic compound of other pheromones (Rudinsky et al., 1974; Zhang et al., 2009; Liu et al., 2013; Sun et al., 2013).

On the other side, the absence of Darm-CYP6DJ1 in *D. armandi* indicates that isoforms of subgroup Y evolved in all *Dendroctonus*-species after divergence from this species. The isoforms which clustered in this subgroup Dadj/Dfro/Dmex-CYP6DJ(Y)1 and Dpon/Drhi/Dval- CYP6DJ1(Y), are duplicates of subgroup X and reinforce the original function of the CYP6DJ2 subfamily. In addition, the retention of this duplicate over time is also indicative that their duplication is an adaptive advantage for these bark beetles in the detoxification process.

The heme group from Dpon-CYP6DJ1(Y) showed no interaction with the tested monoterpenes, which explains why this isoform recorded no functional activity in the enzymatic assays performed with monoterpenes (+)- and (−)-*α*-pinene, (+)- and (−)-*β*-pinene, *R*-(+)-limonene, (+)-3-carene, myrcene, and *β*-phellandrene, but metabolized the cyclic monoterpene terpinolene, diterpenes (+)-(4*R*)-limonene, and (−)-(4*S*)-limonene (Chiu et al., 2019b). A detailed analysis of amino acid sequences from Dpon-CYP6DJ1 and Dpon-CYP6DJ2 revealed one change in position 222, where a SER residue in CYP6DJ1 had been replaced by PHE in CYP6DJ2. This change might apparently be responsible for the differential activity towards monoterpenes. This type of functional divergence has also been reported in paralogous CYP6AE19 and CYP6AE20 from *H. armigera*, where the VAL residue changes to MET in position 318 in the SRS4, causing the recognition and metabolism of xanthotoxin by CYP6AE (Shi et al., 2022).

### 4.4 Functional divergence

It has been hypothesized that genetic duplication plays an important role in functional diversity, where different selective pressures acting on different nucleotide sites of duplicate genes constrain its function, especially in motifs and functional domains (Fonseca et al., 2007). Our results suggest that the CYP6DE have experimented both type-I and type-II functional divergence, and the CYP6DJ only type-I across the evolutionary history from *Dendroctonus* spp. Type-I functional divergence has occurred in both subfamilies, but the amino acid patterns in phylogenetic subgroup A from the CYP6DE subfamily is more conserved compared to that in subgroups B and C. This might explain the retention of subgroup A in all analyzed *Dendroctonus* species (plesiomorphic condition), and the gain or loss of isoforms from subgroup B or C (apomorphic condition). The type-II functional divergence in CYP6DE subgroups is explained by the changes from polar to non-polar amino acids. In both types of functional divergence, the changes are concentrated (182 and 11 amino acid residues type-I and type II, respectively) into regions SRS2, SRS3, SRS4, and K-helix, except 43 residues of type-II functional divergence that were not located in some domain or motif. These findings suggest that residues of apomorphic isoforms from subgroups B and C possess more versatility than residues of subgroup A isoforms, and perhaps also more specificity, with respect to the monoterpenes that can be metabolized.

In the case of the CYP6DJ subfamily, subgroup X from the CYP6DJ1 isoform is the plesiomorphic condition in these bark beetles, and is more conserved compared to the apomorphic subgroup Y. It is interesting to know that subgroup X has been lost in *D. mexicanus*. A detailed analysis of the transcriptome of this species showed that isoforms of this group are not present; yet it is necessary to confirm this result by analyzing sibling species, *D. vitei*, or a second transcriptome of that species.

Thereby, given the low residues number from CAASs (4), which did not fall in some domain or motif, we though that CYP6DE and CYP6DJ subfamilies have evolved under different selective pressures and functional constraints linked with monoterpenes detoxification through the evolutionary history of *Dendroctonus*. The fact that different subgroups are present in each species supports the birth-death model evolution of CYP genes which is the result of gene duplication and mutational changes. We recognized that subgroups B, C and Y originated as duplicates from the ancestral subgroups A and X, represent functional reinforcements of the detoxification process and could be act as an adaptive advantage in these bark beetles. In addition, our evidence suggest that these cytochromes can transform all assayed monoterpenes, but that some isoforms might preferentially metabolize some compounds and produce compounds that can act as pheromones or synergistic compounds in some species. Experimental evidence is required to confirm the activity of some isoforms from the subfamilies analyzed.

## Data Availability

The datasets presented in this study can be found in online repositories. The names of the repository/repositories and accession number(s) can be found in the article/Supplementary Material.
