# Supplementary material for: Evolution and functional role prediction of the CYP6DE and CYP6DJ subfamilies in Dendroctonus (Curculionidae: Scolytinae) bark beetles
Source: Front Mol Biosci. 2023 Oct 9;10:1274838. doi: 10.3389/fmolb.2023.1274838 (PMC10593416; doi:10.3389/fmolb.2023.1274838)
Supplement: Supplementary file 6 [file Datasheet2.DOCX]

Evolution and functional role prediction of the CYP6DE and CYP6DJ subfamilies in Dendroctonus (Curculionidae: Scolytinae) bark beetles

J. Manuel Quijano-Barraza^1^, G. Zúñiga^1^, Claudia Cano-Ramírez^1*^, María Fernanda López^1*^ Gema L. Ramírez-Salinas^2^ and Moises Becerril^1^.

^1^Laboratorio de Variación Biológica y Evolución, Departamento de Zoología, Escuela Nacional de Ciencias Biológicas, Instituto Politécnico Nacional, Prolongación de Carpio y Plan de Ayala s/n, Miguel Hidalgo, México City, CP 11340, México

^2^Laboratorio de Modelado Molecular y Diseño de fármacos, Departamento de Bioquímica, Escuela Superior de Medicina, Instituto Politécnico Nacional, México City, CP 11340, México

*** Correspondence:**
**Claudia Cano Ramírez**

[clacanram@yahoo.com](mailto:clacanram@yahoo.com)

María Fernanda López

[mariferlopez@hotmail.com](mailto:mariferlopez@hotmail.com)


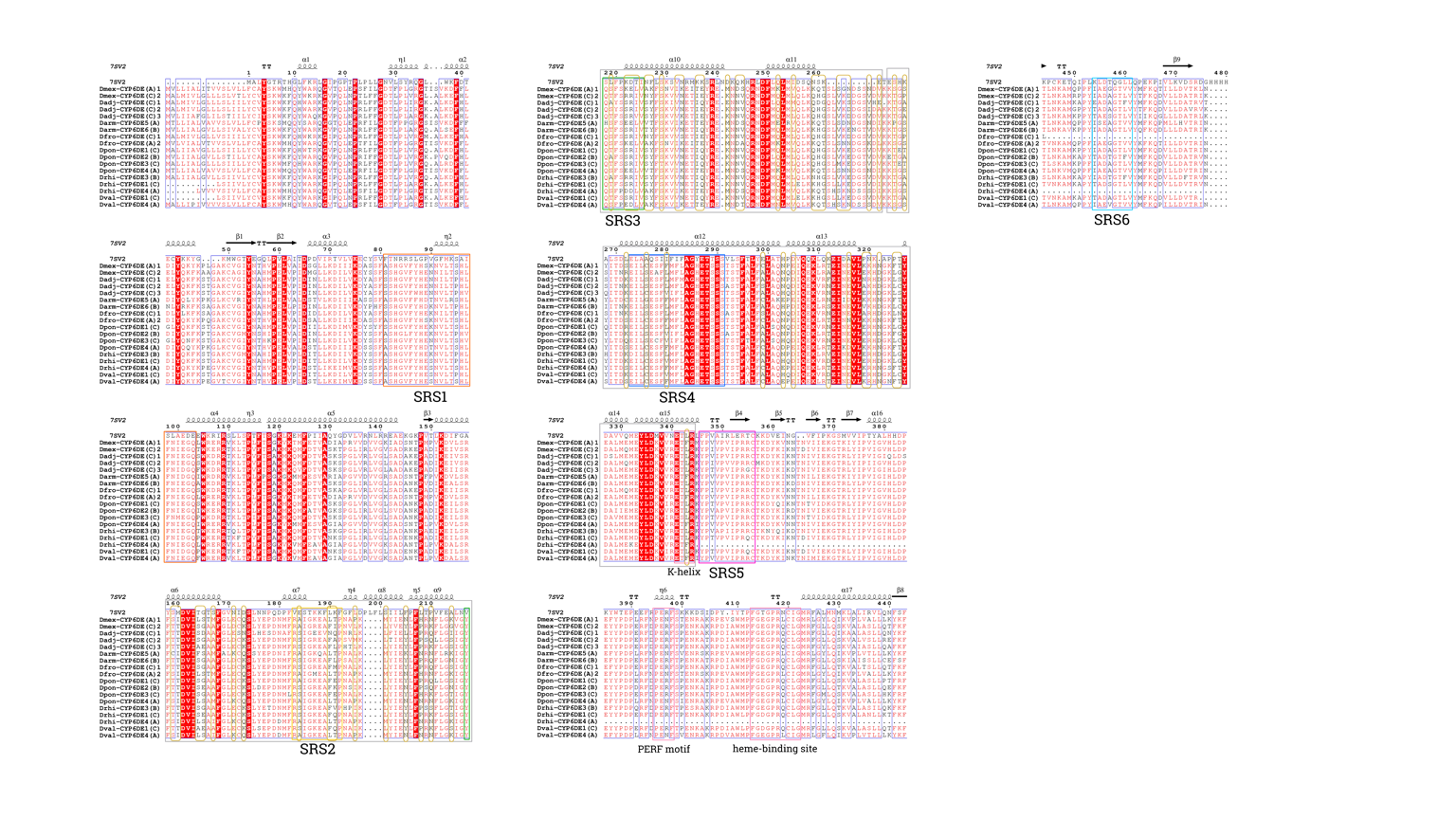


**Supplementary Figure 1.** Multiple alignment of amino acid sequences of CYP6DE subfamily from some species of *Dendroctonus* genus, based on the crystal structure CYP3A5 from *Homo sapiens* (PDB ID:7SV2). Six substrate recognition sites are delimited in boxes: SRS1- orange, SRS2-yellow, SRS3-green, SRS4-blue, SRS5-magenta, SRS6-cyan). CYP motifs are indicated in boxes of pink color. Type I and type II critical amino acid sites (CAASs) from the functional divergence analysis are indicated in boxes of silver and gold color, respectively. The helices are marked as a or b, based on the automatic assignment performed in ESPript 3.0 software, according to the template of the CYP3A5 protein structure.


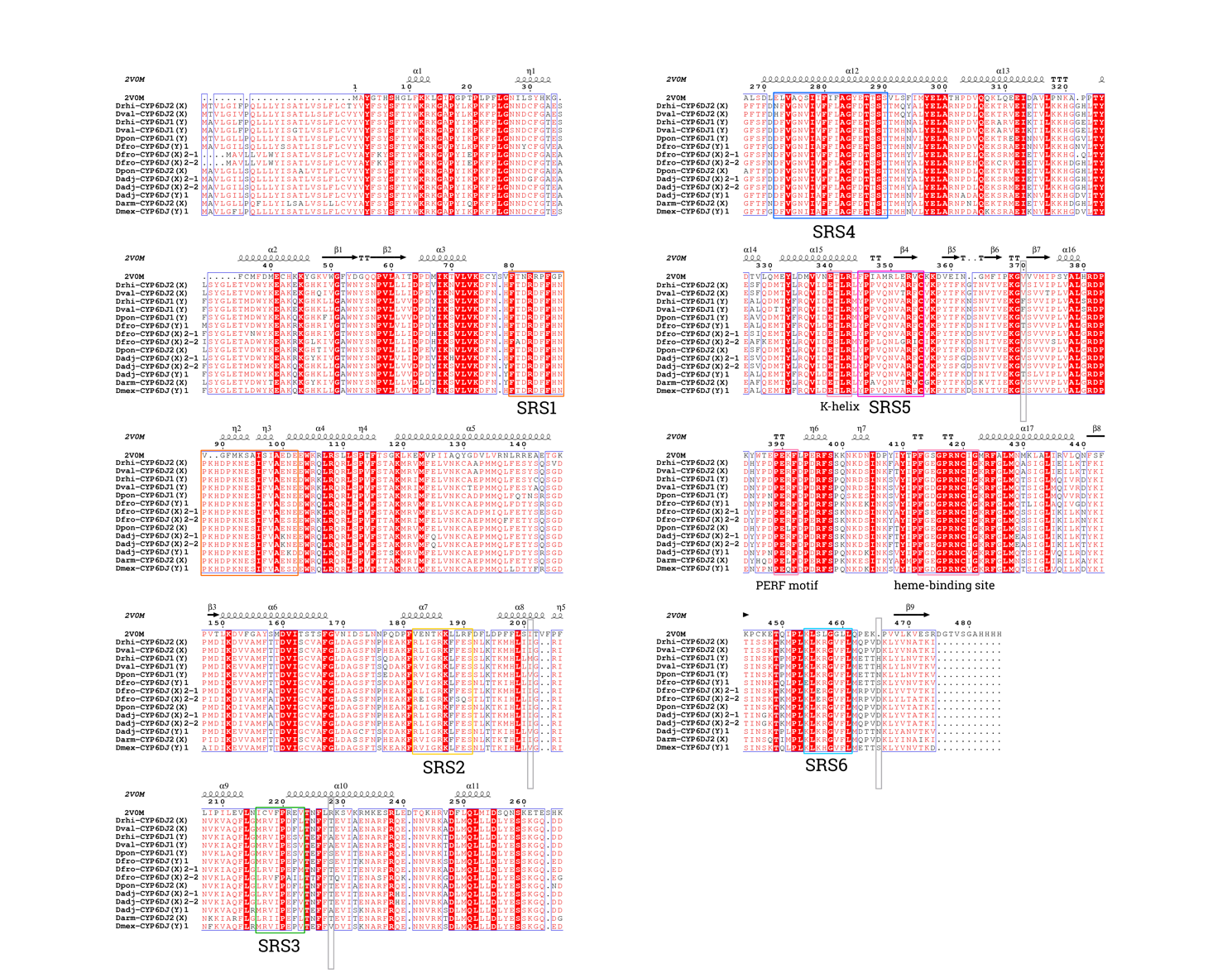


**Supplementary Figure 2**. Multiple alignment of amino acid sequences of CYP6DJ subfamily from some *Dendroctonus* species, based on the crystal structure CYP3A4 from *Homo sapiens* (PDB ID:2V0M). Six substrate recognition sites are delimited in boxes: SRS1- orange, SRS2- yellow, SRS3- green, SRS4- blue, SRS5- magenta, SRS6- cyan. CYP motifs are represented in boxes of pink color. Type I and type II critical amino acidic sites (CAASs) from the functional divergence analysis are indicated boxes in silver and gold color, respectively. The helices are marked as a or b based on the automatic assignment performed in the ESPript 3.0. software, according to the template of the CYP3A4 protein structure

**Supplementary Figure 3**. Structure match of the CYP6DE and CYP6DJ subfamilies (gray color) with the highest and the lowest TM-Score versus the template structure. A. Dfro-CYP6DE2 and B. Darm-CYP6DE5 overlapped with human CYP3A5 isoform (7sv2). C. Darm-CYP6DJ2 and D. Dpon-CYP6DJ1 with human CYP3A4 isoform (2v0m).
