# Supplementary material for: Evolution and functional role prediction of the CYP6DE and CYP6DJ subfamilies in Dendroctonus (Curculionidae: Scolytinae) bark beetles
Source: Front Mol Biosci. 2023 Oct 9;10:1274838. doi: 10.3389/fmolb.2023.1274838 (PMC10593416; doi:10.3389/fmolb.2023.1274838)
Supplement: Supplementary file 8 [file Datasheet1.DOCX]

Figure Captions and Supplementary Material

**1. FIGURES CAPTIONS**

**FIGURE 1**. Phylogeny obtained of some CYP6 family proteins based on a Bayesian inference analysis. The subgroups from the CYP6DE subfamily are indicated as “A”, “B”, “C”, and the subgroups belonging to the CYP6DJ subfamily are marked as “X” and “Y”. Priors used were BLOSUM62 as the best amino acid substitution model and a birth-death model. The numbers indicate Bayesian posterior probabilities.

**FIGURE 2**. Mapping of the CYP6DE and CYP6DJ phylogenetic subgroups over a schematic representation of the *Dendroctonus* genus, the phylogenetic sub-groups are indicated with colored dots over both the terminals and common ancestor branches when at least one isoform from the corresponding subgroup is present.

**FIGURE 3**. Phylogenetic reconciliation analysis of *Dendroctonus*-species versus CYP6DJ and CYP6DE subfamilies isoforms. (A). Minimum instability clades of CYP6DE subfamily isoforms in marked in color. B) Minimum instability clades of CYP6DJ subfamily isoforms marked in color.

**FIGURE 4**. Most stable interactions in the molecular docking between monoterpenes and isoforms from phylogenetic subgroup A. Isoforms are represented in gray, the catalytic site in blue-orange and most important interacting residues from SRSs with specific monoterpenes (green) in different color**.** (A) Dval-CYP6DE4 c. (-)-ß -pinene, ΔG=-25.79 kJ; (B) Drhi-CYP6DE4 c. (+)-ß-pinene, ΔG=-23.06 kJ; (C) Dmex-CYP6DE1 c. ß-Myrcene, ΔG=-25.03 kJ; D) Dfro-CYP6DE2 c. (-)- a-Pinene, ΔG=-25.07 kJ; (E) Dpon-CYP6DE4 c. (-)- a -Pinene, ΔG=-25.95 kJ; (F) Darm-CYP6DE5 c. (-)-ß-pinene, ΔG=-24.53 kJ; (G) Dfro-CYP6DE2 c. (-)-ß-pinene, ΔG=-25.07 kJ.

**FIGURE 5**. Most stable interactions in the molecular docking between monoterpenes and isoforms from phylogenetic subgroup B. Isoforms are represented in gray, the catalytic site in blue and most important interacting residues from SRSs with specific monoterpenes (green) in different color. (A) Dpon-CYP6DE2 c. (-)-ß-pinene, ΔG=-24.45 kJ; (B) Darm-CYP6DE6 c. (+)-a-pinene, ΔG=-25.16 kJ; (C) Drhi-CYP6DE3 c. (+)-a-pinene, ΔG=-24.66 kJ.

**FIGURE 6**. Most stable interactions in the molecular docking between monoterpenes and isoforms from phylogenetic subgroup C. Isoforms are represented in gray, the catalytic site in blue-orange and most important interacting residues from SRSs with specific monoterpenes (green) in different color**.** (A) Dpon-CYP6DE1 c. (+)-a-pinene, ΔG=-24.82 kJ; (B) Dpon-CYP6DE3 c. (+)-ß-pinene, ΔG=-23.19 kJ; (C) Dfro-CYP6DE1 c. (-)-ß-pinene, ΔG=-24.66 kJ; D) Dadj-CYP6DE1 c. (-)-ß-pinene, ΔG=-24.82 kJ; (E) Dadj-CYP6DE2 c. (+)-a-pinene, ΔG=-25.16 kJ; (F) Dadj-CYP6DE3 c. (+)-ß-pinene, ΔG=-23.61 kJ.

**FIGURE 7**. Most stable interactions in the molecular docking between monoterpenes and isoforms from phylogenetic subgroup X. Isoforms are represented in gray, the catalytic site in blue-orange and most important interacting residues from SRSs with specific monoterpenes (green) in different color. (A) Dadj-CYP6DJ2-1 c. (+)-ß-pinene, ΔG=-27.92 kJ; (B) Dadj-CYP6DJ2-2 c. (-)-a-pinene, ΔG=-26.87 kJ; (C) Dfro-CYP6DJ2-1 c. (-)-a-pinene, ΔG=-26.83 kJ; D) Darm-CYP6DJ2 c. (-)-ß-pinene, ΔG=-26.04 kJ; (E) Dfro-CYP6DJ2-2 c. (-)-a-pinene, ΔG=-26.46 kJ; (F) Dfro-CYP6DJ2-1 c. (+)-ß-pinene, ΔG=-26.83 kJ; (G) Dpon-CYP6DJ2 c. (-)-a-pinene, ΔG=-27.13 kJ; Drhi-CYP6DJ2 c. (-)-a-pinene, ΔG=-27.04 kJ; Dval-CYP6DJ2 c. (+)-ß-pinene, ΔG=-26.79 kJ.

**FIGURE 8**. Most stable interactions in the molecular docking between monoterpenes and isoforms from phylogenetic subgroup Y. Isoforms are represented in gray, the catalytic site in blue-orange and most important interacting residues from SRSs with specific monoterpenes (green) in different color. (A) Dfro-CYP6DJ1 c. (+)-ß-pinene, ΔG=-24.15 kJ; (B) Dadj-CYP6DJ1 c. (+)-a-pinene, ΔG=-24.2 kJ; (C) Dmex-CYP6DJ1 c. (-)-a-pinene, ΔG=-24.47 kJ; D) Drhi-CYP6DJ1 c. (+)-ß-pinene, ΔG=-26.37 kJ; (E) Dval-CYP6DJ1 c. (+)-a-pinene, ΔG=-27.08 kJ; (F) Dval-CYP6DJ1 c. (+)-ß-pinene, ΔG=-27.08 kJ.

**2 SUPPLEMENTARY MATERIAL CAPTIONS**

**Supplementary Table 1**. Accession number, codes of the CYP6, CYP9, CYP345 families from several species coleopteorans used in this study.

**Supplementary Table 2**. Templates used for the modelling of the CYP6DE and CYP6DJ isoforms and the corresponding ERRAT scores and Ramachandran plot allowed angles (%).

**Supplementary Table 3**. Physicochemical properties, signal peptide and predicted sub-cellular location of the isoforms CYP6DE and CYP6DJ subfamilies.

**Supplementary Table 4**. Phylogenetic instability results of the CYP6DE and CYP6DJ subfamilies.

**Supplementary Table 5**. Molecular docking results of the CYP6DE and CYP6DJ subfamilies isoforms with the monoterpenes (+)- and (-)-*α*-pinene, (+)- and (-)-*β*-pinene, (+)-3-carene, *R*-(+)-limonene, and myrcene.

**Supplementary Table 6**. Functional divergence results of the CYP6DE and CYP6DJ subfamilies.

**
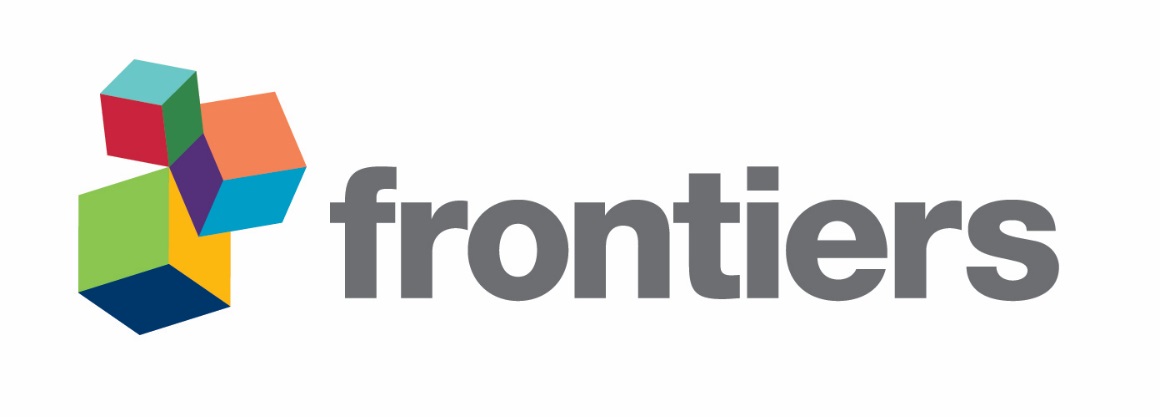
**
